# *OsABCG15* encodes a membrane protein that plays an important role in anther cuticle and pollen exine formation in rice

**DOI:** 10.1007/s00299-014-1666-8

**Published:** 2014-08-20

**Authors:** Lina Wu, Yusheng Guan, Zigang Wu, Kun Yang, Jun Lv, Richard Converse, Yuanxin Huang, Jinxiong Mao, Yong Zhao, Zhongwei Wang, Hengqi Min, Dongyang Kan, Yi Zhang

**Affiliations:** 1 College of Agronomy and Biotechnology, Southwest University, Chongqing, 400715 China; 2 Engineering Research Center of South Upland Agriculture, Ministry of Education, Southwest University, Chongqing, 400715 China; 3 Cincinnati State Technical and Community College, 3520 Central Parkway, Cincinnati, OH 45223 USA; 4 Nanchong Academy of Agricultural Sciences, Nanchong, 637000 Sichuan China

**Keywords:** ABC transporter, Rice, Anther cuticle, Pollen exine, Pollen development

## Abstract

*****Key message***:**

**An ABC transporter gene (** ***OsABCG15*** **) was proven to be involved in pollen development in rice. The corresponding protein was localized on the plasma membrane using subcellular localization.**

**Abstract:**

Wax, cutin, and sporopollenin are important for normal development of the anther cuticle and pollen exine, respectively. Their lipid soluble precursors, which are produced in the tapetum, are then secreted and transferred to the anther and microspore surface for polymerization. However, little is known about the mechanisms underlying the transport of these precursors. Here, we identified and characterized a member of the G subfamily of ATP-binding cassette (ABC) transporters, OsABCG15, which is required for the secretion of these lipid-soluble precursors in rice. Using map-based cloning, we found a spontaneous A-to-C transition in the fourth exon of *OsABCG15* that caused an amino acid substitution of Thr-to-Pro in the predicted ATP-binding domain of the protein sequence. This *osabcg15* mutant failed to produce any viable pollen and was completely male sterile. Histological analysis indicated that *osabcg15* exhibited an undeveloped anther cuticle, enlarged middle layer, abnormal Ubisch body development, tapetum degeneration with a falling apart style, and collapsed pollen grains without detectable exine. *OsABCG15* was expressed preferentially in the tapetum, and the fused GFP-OsABCG15 protein was localized to the plasma membrane. Our results suggested that *OsABCG15* played an essential role in the formation of the rice anther cuticle and pollen exine. This role may include the secretion of the lipid precursors from the tapetum to facilitate the transfer of precursors to the surface of the anther epidermis as well as to microspores.

**Electronic supplementary material:**

The online version of this article (doi:10.1007/s00299-014-1666-8) contains supplementary material, which is available to authorized users.

## Introduction

Sexual reproduction is essential for metagenesis and genetic recombination in flowering plants. In plant gamogenesis pollen grains and the embryo sac are the two important participants. The development of viable pollen grains within the anther is a prerequisite for sexual propagation of flowering plants, and pollen development requires a safe compartment for microgametophytogenesis to proceed correctly. To achieve this, plants have evolved two lipid-soluble barriers, i.e., the anther wall cuticle and the exine forming the outer pollen wall, to protect the microspore/pollen grain from environmental and biological stresses.

Fatty alcohols and their derivatives are major components of the anther cuticle and pollen wall, rich in lipids (Ahlers et al. 1999; Jung et al. 2006; Meuter-Gerhards et al. 1999). The cuticle consists of two types of lipophilic biopolymers: cutin and wax (Samuels et al. 2008; Li and Zhang 2010; Riederer and Muller 2006; Kerstiens 1996). Plant waxes, the major component of the cuticle, predominantly contain alcohols, aldehydes, ketones, alkanes, and esters derived from very-long-chain fatty acids (VLCFAs), with chain length from C20 to C34 (Kunst and Samuels 2003). The insoluble polymer cutin contains hydroxylated and epoxy C16 and C18 fatty acids, which define the framework of the plant cuticle (Kolattukudy 2001; Heredia 2003; Nawrath 2006; Li and Zhang 2010). The highly sculptured exine is mainly composed of the biopolymer sporopollenin, which is thought to be made of phenolics and polyhydroxylated unbranched aliphatics, coupled by ester and ether linkages. (Guilford et al. 1988; Scott et al. 2004; Wiermann et al. 2001). The formation of the pollen exine largely relies on the secretory role of the tapetum (Pacini et al. 1985; Shivanna et al. 1997). Rice (*Oryza sativa*) and many other plants have a secretory tapetum with Ubisch bodies, which are specialized structures forming along the inner surface of the tapetum/anther locule. They transfer lipid-soluble components from the tapetum to the microspores (Huysmans et al. 1998).

Recent forward and reverse genetic studies have verified a number of genes that contribute to epicuticular wax and pollen exine synthesis and deposition. For example, *DEX1*, *NEF1* and *RPG1* are involved in exine pattern formation in Arabidopsis, and no primexine will develop if these genes mutate (Paxson-Sowders et al. 2001; Ariizumi et al. 2004; Guan et al. 2008; Ariizumi and Toriyama 2011). Other genes like *MS2*, *AC*OS *5*, and *CYP703A2* (Ariizumi and Toriyama 2011; Zhang et al. 2011) encode enzymes related to the synthesis of sporopollenin precursors, and their mutations always result in a smooth pollen surface with a complete absence of exine. Mutations of similar genes in rice show additional abnormalities, including epidermal smoothing of the anther and/or missing Ubisch bodies. Such genes include *WDA1*, *CYP703A3*, *CYP704B2*, and *DPW*, which encode lipid-soluble metabolic products (Jung et al. 2006; Aya et al. 2009; Li et al. 2010; Shi et al. 2011). These defects suggest that most of these rice genes participate in the synthesis of precursors for pollen, exine sporopollenin and anther epicuticle (Li et al. 2010; Ariizumi and Toriyama 2011; Kandel et al. 2006). For instance, *CYP704B2*, the rice ortholog of *CYP704B1* (a cytochrome P450 family member), is conserved among terrestrial plants and is preferentially expressed in tapetal cells. The recombinant CYP704B2 protein can catalyze the hydroxylation of palmitic acid and unsaturated C18 fatty acids in the ω position of the carbon chain. The *cyp704B2* mutant shows a defective anther epidermal cuticle and aborted pollen grains without obvious exines (Li et al. 2010), which indicates that *CYP704B2* controls a conserved biosynthetic pathway for the biopolymers sporopollenin and cutin.

The above-mentioned genes related to lipid-soluble precursor synthesis are mainly expressed in tapetal cells, but the biopolymers are found largely on the surface of the outer layer of the anther wall and pollen walls. After production, these precursors must be secreted from the tapetum and transferred onto the wall surfaces to be polymerized into biopolymers of sporopollenin, wax, and cutin. Based on their expressions in the tapetum and other molecular features, some ATP-binding cassette (ABC) transporters and lipid transfer proteins (LTPs) are proposed to play important roles in the secretion and the transfer processes, respectively (Ariizumi and Toriyama 2011). However, relatively little has been characterized regarding these two types of transport genes in pollen development.

ABC transporters are a large family of proteins found in bacteria, yeast, plants, animals and humans. They are known for their ability to transport a broad range of substances across biological membranes using energy released by ATP hydrolysis. These transporters have been largely studied in animals, because many are involved in multidrug resistance, which limits the long-term use of drugs for treatment of chronic diseases (Borowski et al. 2005). Many ABC transporters are also found in plants, and the ABCG/WBC (white–brown complex homolog) subfamily is the largest of the plant ABC subfamilies. ABCG transporters have a nucleotide binding domain (NBD) and a transmembrane domain (TMD) in a family-defining reverse ABC transporter topology (NBD-TMD). They are important for the export of wax and cutin monomers (Bessire et al. 2011). Thus far, about 10 plant ABCG genes have been described, including *AtABCG11/WBC11/COF1*, *AtABCG12/WBC12/CER5* (Pighin et al. 2004; Bird et al. 2007; Panikashvili et al. 2007; Ukitsu et al. 2007), *AtABCG13/WBC13* (Panikashvili et al. 2011), *AtABCG19/WBC19* (Mentewab and Stewart 2005), *AtABCG25/WBC26* (Kuromori and Shinozaki 2010; Kuromori et al. 2010), *AtABCG26/WBC27*(Xu et al. 2010; Quilichini et al. 2010; Choi et al. 2011; Dou et al. 2011), *AtABCG32/PDR4/PEC1* (Bessire et al. 2011), and *Hv/OsABCG31* (Chen et al. 2011). Almost all of these ABCG transporters are located on the plasma membrane and, with the exceptions of ABCG19 and 25, are required by the lipid-soluble precursors for extracellular secretion from the epidermis and/or tapetal cells. Functional loss of these lipid-transporting genes always results in defects in epicuticle and/or exine formation and eventually leads to abnormal phenotypes such as wilt, organ fusion, and sterility in the mutant plant. Remarkably, ABCG26/WBC27 was well characterized in Arabidopsis by three independent groups during the same period (Dou et al. 2011; Choi et al. 2011; Quilichini et al. 2010). *AtABC26/WBC27* was especially expressed in the tapetum of early anthers and the protein was localized to the plasma membrane. The *atabcg26/wbc27* mutants are unable to export lipid-soluble precursors from the tapetum and also lack exine, experience pollen degeneration, and suffer a drastic decrease in seed setting. These results indicated that ABCG26/WBC27 plays an important role in sporopollenin precursor transport from the tapetum to the microspore.

More than 130 ABC transporters are known in rice, and 50 of them belong to the ABCG sub-family (Verrier et al. 2008). Their functions remain largely unknown. OsABCG15 (Verrier et al. 2008) is the ortholog of AtABCG26/WBC27 and has been reported to play a role in microspore development (Qin et al. 2013; Zhu et al. 2013; Niu et al. 2013) Here, we provide more detailed information on the defects resulting from a mutation of A-to-C transition in the fourth exon of *OsABCG15*; this evidence strongly suggests that OsABCG15 is a plasma-membrane localized protein. Unlike previous reports, we also found that the surfaces of the connective tissue, including stomata, failed to include lipid materials. Also, the successive and centrifugal growth of wax on wild-type anther epidermis is reported. As in previous reports on this gene, the male-sterile *osabcg15* displayed defects such as undeveloped anther epidermal cuticle, absence of Ubisch bodies, lack of exine, and completely collapsed microspores. Consistent with the lipid transport function in the outer layers of the anther and pollen walls, the *OsABCG15* is strongly expressed in the tapetum of young anthers and its product is localized to the plasma membrane.

## Materials and methods

### Mutant materials and growth conditions

The mutant in this study was isolated from the offspring of an *Oryza sativa indica* restorer line, Jinhuiyihao, as described previously (Zhang et al. 2008b). To further characterize the defects of this mutant, a near-isogenic line (NIL) of male sterility (*osabcg15* in this report) was bred by backcrossing an *indica* maintainer line, II-32B, as a recurrent parent (Zhang et al. 2008b). For a higher efficiency of transformation in complementation testing, we bred another NIL of male sterility by backcrossing with Nipponbare (*O. sativa japonica*) as a recurrent parent. All rice plants were grown in the paddy field of Southwest University, Beibei, Chongqing, China (29°49′18′′N 106°25′45′′E). The F_2_ mapping population was generated from a cross between the *osabcg15* mutant (*indica*) and Nipponbare (*japonica*). In the F_2_ population, male-sterile plants were selected for gene mapping.

### Characterization of the mutant phenotype

Mature stage plants and flowers at anthesis were photographed with an Olympus C-770 digital camera (Tokyo, Japan). Mature anthers were examined using a Nikon SMZ1500 stereoscope and photographed with a Nikon DS-5Mc digital camera (Tokyo, Japan). The wild-type and *osabcg15* mutant anthers were separately crushed and stained in 1 % I_2_–KI solution for 3–5 s to dye starch then photographed with a Nikon E600 microscope. Observation of anther development by semi-thin sections and transmission electron microscopy (TEM) was performed as described by Li et al. (2006) using an Hitachi H-7500 transmission electron microscope (Tokyo, Japan). Bright-field photographs of anther cross-sections were taken under a Nikon E600 microscope using a Nikon DS-5Mc digital camera, and the TEM results were photographed using a Gatan 832 CCD camera (Pleasanton, CA, USA). For scanning electron microscopy (SEM) observations, anthers were collected and processed essentially as described by Keijzer et al. (1996) and observed with a Quanta 200 scanning electron microscope (FEI, Hillsboro, OR, USA) under a strong vacuum. Anthers from different developmental stages, as defined by Zhang et al. (2011), were collected based on spikelet length and lemma/palea morphology.

### Map-based cloning of the *OsABCG15* gene

The *OsABCG15* locus was first mapped between two simple sequence repeat (SSR) markers, RM7434 and RM275, on chromosome 6 (Zhang et al. 2008b). For fine mapping, 2,157 sterile individuals from the F_2_ generation were analyzed with a set of linked primers (see Supplementary Table 1 online for sequences). *OsABCG15* was finally defined between two of these markers, Chr6STS20 and Chr6STS16, within a 2.2-kb region (Fig. 5). The PCR products were separated on 10 % polyacrylamide gels, and bands were visualized by a silver-staining method (Zhang et al. 2008b).

### Complementation of the *osabcg15* mutant

For functional complementation of the rice *osabcg15* mutant, a genomic fragment of ~8.5 kb containing the entire *OsABCG15* coding region, a 1,596-bp upstream sequence, and a 1,060-bp downstream sequence was digested from the Nipponbare bacterial artificial chromosome (BAC) (OSJNBa0005N07r) with *Eco*RI and *Sbf*I and subcloned into the binary vector *pCAMBIA1301* with a hygromycin resistance marker to generate the *p1301*-*OsABCG15* construct. Calli induced from homogenous *osabcg15* young panicles were used for transformation with *Agrobacterium tumefaciens* LBA4404 carrying the *p1301*-*OsABCG15* plasmid. The positive transformed plant was identified by GUS assay. To confirm whether or not the fertility of *osabcg15* was restored by *OsABCG15*, the pollen grains of transgenic lines of T_0_ were assayed by I_2_–KI staining and observed under a light microscope, and the co-separation of GUS coloration and fertility in the T_1_ generation transgenic line was also investigated.

### Phylogenetic analysis

The full-length amino acid sequence of OsABCG15 and the 25 most similar sequences identified via a BLAST search were aligned with the ClustalW tool using the default parameters. The alignment (see Supplemental Fig. 4 online) was used to construct a neighbor-joining tree (Saitou and Nei 1987) in MEGA 5 (Tamura et al. 2011) using the following parameters: Poisson model, complete deletion, and 1,000 bootstrap replicates.

### Quantitative RT-PCR assay

Total RNA was isolated using TRIzol reagent (Invitrogen, Carlsbad, CA, USA), as described by the supplier, from rice tissues, including sheath, blade, stem, root, seed, and anther at different developmental stages. The anther stages were based on spikelet length and anther morphology. The first strand of complementary DNA was synthesized from 1 μg of total RNA using oligo(dT) primers in a 25-μL reaction volume using the RNA PCR Kit (AMV) ver. 3.0 (TaKaRa, Otsu, Japan). RT-qPCR was performed on a StrataGene Mx3000p detection system using SYBR Green I (TaKaRa). All PCR experiments were conducted using 40 cycles of 95 °C for 30 s, 60 °C for 30 s, and 72 °C for 30 s, in a reaction mixture containing 10 pmol of each primer, 3 mM MgCl_2_, and a 1:10 dilution of each cDNA pool (per biological replicate) as a template. All reactions were performed in triplicate, with *ACTIN* as the normalized reference gene for all comparisons. The primers for RT-qPCR are listed in Supplementary Table 1 online.

### In situ hybridization

Wild-type spikelets of different developmental stages were fixed in 5 % acetic acid, 50 % ethanol, and 3.7 % formaldehyde in water for 16 h at 4 °C. They were dehydrated through an ethanol series, embedded in Paraplast Plus (Sigma-Aldrich, St. Louis, MO, USA), and sectioned at 8 µm using a Leica RM2245 rotary microtome (Nussloch, Germany). The 323-bp gene-specific *OsABCG15* antisense and sense probes were amplified with the primers antisense F/R and sense F/R in situ and labeled using the DIG RNA Labeling Kit (SP6/T7) (Roche, Penzberg, Germany) according to the vendor’s recommendations. Pretreatment of sections, hybridization, and immunological detection were performed as described previously (Xiao et al. 2009). The primer sequences are listed in Supplementary Table 1.

### OsABCG15 membrane localization analysis

To determine whether OsABCG15 was localized on the plasma membrane, we generated a GFP-OsABCG15 fusion protein expression construct under the control of the 35S promoter. The green fluorescent protein (GFP) fragment obtained from the plasmid *pUCLNGFP2* (a gift from Prof De Ye, College of Biological Science, China Agriculture University, Beijing, China) was inserted into *Xba*I/*Sac*I-digested *pCAMBIA 1300221* (also a gift from De Ye) to make *p35S*-*GFP*. The *OsABCG15* cDNA was amplified from mixed cDNA and reverse transcribed from total RNA from young inflorescences of Nipponbare with the GFP fusion F/R primers (Supplementary Table 1 online). The PCR product was digested by *Bgl*II/*Kpn*I and subsequently cloned into the *Bgl*II/*Kpn*I-digested *p35S*-*GFP* vector to generate *p35S*-*GFP*-*OsABCG15.* The construct was introduced into *Nicotiana benthamiana* epidermal cells by *Agrobacterium* infiltration. Cells with GFP fluorescence were observed under a fluorescence confocal microscope (Olympus FV1000-MPE).

## Results

### Isolation and characterization of the *osabcg15* mutant phenotypes

In 2008, we reported a spontaneous male-sterile mutant *ostd* (*t*) isolated from the offspring of the *indica* restorer line Jinhuiyihao. Its male sterility of the mutant was not sensitive to changes in temperature and photoperiod and was controlled by a single recessive gene that mapped between the SSR markers RM7434 and RM275 on chromosome 6. To classify the exact sterility of the mutant, a near-isogenic line (NIL) of male sterility was bred by backcrossing an *indica* maintainer line, II-32B, as a recurrent parent (Zhang et al. 2008b). Since the protein encoded by the target gene is an ABC transporter of *OsABCG15*, we renamed this male-sterile NIL *osabcg15* and the gene *OsABCG15*.

**Fig. 1:**
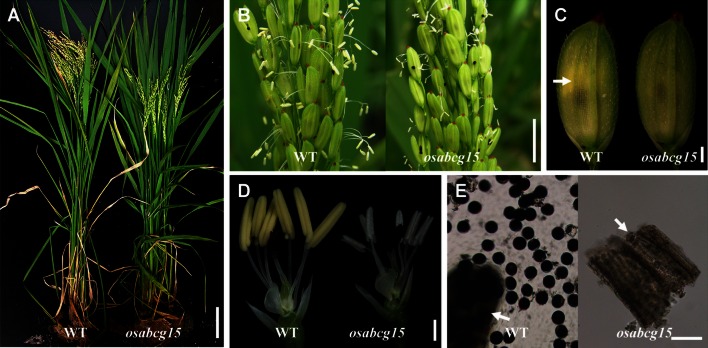
Phenotype comparison between wild type and *osabcg15*. Wild type (*left*) and *osabcg15* (*right*) are compared **a** during ripening, **b** when dehiscing, **c** before anthesis, and **d** floral organs after removal of the palea and lemma. *Arrows* in **c** indicate *yellow* anthers below the palea of the wild-type flower. **e** I_2_–KI staining of pollen grains showing ample black-dyed fertile pollen in the wild-type plants (*left*) and anther residues but no pollen but in the *osabcg15* (*right*) mutant. *Arrows* indicate anther residues produced by crushing anthers in I_2_–KI solution. *WT* wild type. *Bars* 10 cm in **a**, 1 cm in **b**, 1 mm in **c**) and **d**, and 100 µm in **e**

There were no differences between the wild-type II-32B and its sterile counterpart, NIL *osabcg15*, in vegetative and floral development. However, unlike the wild type, *osabcg15* failed to produce any viable pollen grains and thus set no seed (Fig. 1a). Compared to the yellow, normal-sized anthers of wild-type plants, the mutant anthers were shorter, narrower, and white (Fig. 1b–d). Under a light microscope, there were numerous normal-sized, black-stained pollen grains in wild-type anthers crushed in an I_2_–KI solution, but only anther residues were found in *osabcg15* and no pollen (Fig. 1e).

### Defects of anther wall and pollen exine development in *osabcg15*

Transverse sections of anther were further examined to determine the morphological defects in the *osabcg15* mutant. Based on cellular events visible under a light microscope and a recent classification of anther development (Zhang et al. 2011), rice anther development was classified into 14 stages in this study.

**Fig. 2:**
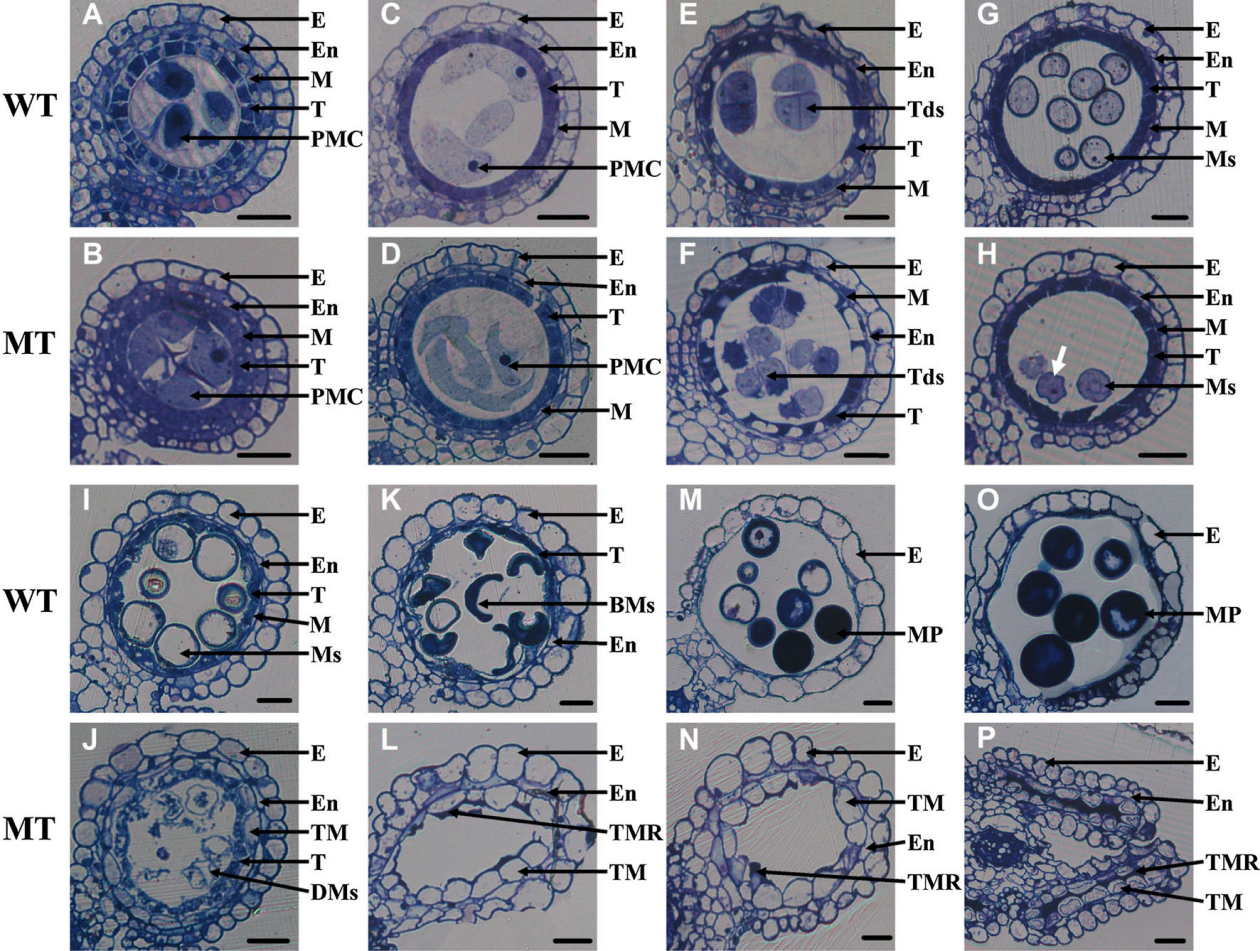
Comparison of transverse sections of wild-type and *osabcg15* anthers. *Upper panels* (**a**, **c**, **e**, **g**, **i**, **k**, **m**, **o**) show anther sections of the wild type, and *lower panels* (**b**, **d**, **f**, **h**, **j**, **l**, **n**, **p**) show the mutant anther sections. *BMs* binuclear microspores, *DMs* degenerated microspores, *E* epidermis, *En* endothecium, *M* middle layer, *MP* mature pollen, *Ms* microspore, *PMC* pollen mother cells, *T* tapetum, *Tds* tetrads, *TM* thickened middle layer, *TMR* tapetum and microspore residues, *bars* 20 μm. **a**, **b** Stage 6. No difference between the wild type and *osabcg15.* **c**, **d** Stage 8a. No obvious difference between the wild type and *osabcg15*. **e**, **f** Stage 8b. Microspore wrinkling is more pronounced in *osabcg15* tetrads than in wild type. **g**, **h** Stage 9. During tetrad release, some microspores (*arrows*) have persistent wrinkling in the *osabcg15* locule. **i**, **j** Stage 10. Microspores have begun degrading and the middle-layer cells are enlarged in *osabcg15*. **k**, **l** Stage 11. Locules of *osabcg15* plants are almost empty owing to complete degeneration of microspores, and the enlargement of the middle cell layer is more obvious. **m**, **n** Stage 12. The locule of *osabcg15* is empty and the middle-layer cells remain enlarged. **o**, **p** Stage 13. Locule of *osabcg15* is wrinkled

Between wild-type II-32B and *osabcg15* anthers, there were no detectable differences until anther stage 8b (tetrad stage). The pollen mother cells (PMC) as well as the four layers of epidermis, endothecium, middle layer, and tapetum developed normally in wild-type and in *osabcg15* anthers at stage 6 (Fig. 2a, b). As found in the wild type, when the anther of *osabcg15* developed to stage 8a, the middle layer became a very thin band and the PMC formed an oblong cell (Fig. 2c, d). However, after stage 8b, *osabcg15* anthers had obvious morphological abnormalities. At stage 8b (Fig. 2e, f) and stage 9 (Fig. 2g, h), a wrinkled microspore was more frequent in *osabcg15* than in wild type, although *osabcg15* tetrads could form on time and microspores were released normally as in the wild type. From stages 8a to 10, the middle layer of the wild-type anther progressively thinned and finally disappeared at stage 11 (Fig. 2e, g, i, k). By contrast, the thin middle layer was maintained until stage 9 in the *osabcg15* mutant and started abnormally widening by stage 10 and continued to enlarge at stage 11 (Fig. 2f, h, j, l). During stage 10, the microspores of wild type began to vacuolate, increasing in volume and becoming round (Fig. 2i), Then they became lens-like binuclear cells at stage 11 (Fig. 2k). Unlike in wild-type anthers, the mutant microspores began degenerating at stage 10 (Fig. 2j) and disappeared almost completely at stage 11, leaving only remnants in the locule (Fig. 2l), although before this point, the *osabcg15* microspores were released from tetrads at stage 9 as expected. Under a light microscope, it seemed that the same degradation of tapetum took placed from stage 8 to 11 in both wild-type II-32B and in *osabcg15* anthers.

Subsequently, at stages 12 and 13, the microspores of wild type were round, became more enlarged and enriched in starch, and developed into mature pollen that filled the locule. Meanwhile, only the epidermis of the four layers in anther wall was retained and the tapetum almost completely degenerated (Fig. 2m, o). However, in *osabcg15*, the locules were mostly empty because of the complete degeneration of microspores. The anther wall retained 2–3 layers because the middle layer enlarged abnormally and the endothecium frequently degenerated incompletely (Fig. 2n, p).

**Fig. 3:**
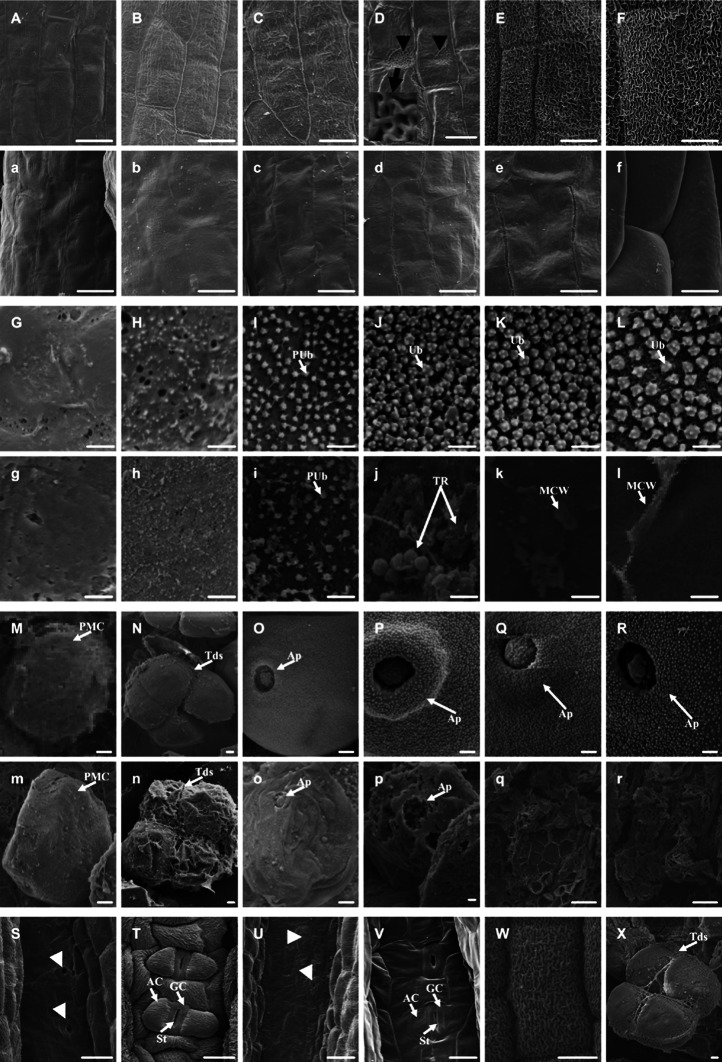
Comparison of the anther and pollen in the wild type and *osabcg15* by scanning electron microscopy. *AC* accessory cell, *Ap* aperture, *GC* guard cell, *MCW* middle layer cell wall, *PMC* pollen mother cell, *pUb* prim-Ubisch, *St* stoma, *Tds* tetrads, *TR* tapetal residues (orbicules), *Ub* Ubisch. *Black* and *white arrowheads* in **D** and **U** indicate the starting points of epidermal sculpture and stomata in the connective tissue, respectively. The *black arrow* in **D** indicates the magnification of the crowded wax crystals. *Bars* 10 μm in **A**–**F**, **W**, and **a**–**f**; 1 μm in **G**–**R**, **S**–**V**, **X** and **g**–**p**; 20 μm in **q** and **r**. Exo-surface of the wild-type anther at stage 7 (**A**), stage 8b (**B**), stage 9 (**C**), early stage 10 (**D**), stage 11 (**E**), and stage 13 (**F**). Exo-surface of the *osabcg15* anthers at stage 7 (**a**), stage 8b (**b**), stage 9 (**c**), early stage 10 (**d**), stage 11 (**e**), and stage 13 (**f**). Ubisch development of the wild-type tapetum at stage 7 (**G**), stage 8b (**H**), stage 9 (**I**), early stage 10 (**J**), stage 11 (**K**), and stage 13 (**L**). Ubisch development of the *osabcg15* tapetum at stage 7 (**g**), stage 8b (**h**), stage 9 (**i**), early stage 10 (**j**), stage 11 (**k**), and stage 13 (**l**). Pollen development of the wild type at stage 7 (**M**), stage 8b (**N**), stage 9 (**O**), early stage 10 (**P**), stage 11 (**Q**), and stage 13 (**R**). Pollen development of the *osabcg15* at stage 7 (**m**), stage 8b (**n**), stage 9 (**o**), early stage 10 (**p**), stage 11 (**q**), and stage 13 (**r**). Stomatal development in the anther connective tissue of the wild type at stage 8 (**S**) and stage 13 (**T**), respectively. Stomatal development in the anther connective tissue of the *osabcg15* cultivar at stage 8 (**U**) and stage 13 (**V**). **W** Hair-like cuticle could form occasionally on the exo-surface of the *osabcg15* anther at stage 13. **X** Exceptionally, microspores in some tetrads of *osabcg15* are smooth at stage 8b

During pollen development, the most obvious defects under a light microscope in *osabcg15* anthers were the middle-layer expansion and microspore degeneration resulting in empty and shriveled locules. To gain more detailed information on the defects in *osabcg15*, anther samples were investigated using SEM (Fig. 3). First, we viewed the wax crystal formation of the outer layer of the anther wall. At stage 7, in both wild type and *osabcg15*, no crystals formation could be seen on the anther exo-surface, and the epidermal cells were smooth and plate-shaped (Fig. 3A, a). Some vermiform wax crystals were released onto the epidermal surface at stages 8–9 (Fig. 3B, C). These crystals tended to crowd randomly on the epidermal surface at stage 10 (Fig. 3D) and subsequently spread across the entire surface of the epidermis, resulting in a thin cuticle layer at stage 11 (Fig. 3E). Finally, this thin layer became a thick, curly hair-like surface at stages 12–14 (Fig. 3F). The crowding of the vermiform crystals appeared to be rare events and occurred only in certain locations, instead of all over the surface. Therefore, the cuticle growth of an epidermis cell is likely to be successive and centrifugal.

The same epidermal events did not occur in *osabcg15* during anther development. The vermiform crystals did not appear and, therefore, did not crowd, while the epidermis remained smooth because of the lack of these wax crystals at all stages (Fig. 3a–f). Remarkably, the hair-like cuticle formed occasionally on a few of the *osabcg15* mutant anthers (Fig. 3W). Interestingly, similar defects in the anther connective tissue of *osabcg15* were observed in the development of epicuticular wax crystals. At stage 8, in both wild type and mutant, the connective tissue and its cells were plain and smooth, and the stomata differentiated normally (Fig. 3S, U). However, at subsequent stages, especially mature ones such as stage 13, an obvious difference was visible. The wild-type connective tissue was lumpy and the stomatal accessory cells were covered with hair-like crystals (Fig. 3T). In contrast, the connective tissue and all cells including stomatal cells were still smooth in *osabcg15* (Fig. 3V).

Ubisch bodies play an important role in exporting materials. Subsequently, we investigated defects in Ubisch body development in *osabcg15*. At stages 7–8, the tapetum surface exhibited no detectible differences between wild type and mutant, and in both, the inner surfaces of the anther were smooth (Fig. 3G–H, g–h). In wild type from stage 9 onwards, an increasing number of poly-chifres Ubisch bodies appeared on the tapetum surface, and they progressively increased in size (Fig. 3I–L). However, compared with the abundant Ubisch bodies emerging in wild type, the *osabcg15* tapetum surface formed fewer and smaller Ubisch bodies at stage 9 (Fig. 3i). Then, these abnormal Ubisch bodies stopped developing and disappeared due to the complete degeneration of tapetum. Some residues of tapetum were visible at stage 10, and only the smooth surface of middle layer cells appeared during the last stages (Fig. 3j–l).

Normal, mature pollen should form a complex exine structure to protect itself from external conditions. Additionally, we scanned the surfaces of meiocytes and microspores in wild type and *osabcg15*. At stage 7, meiocytes of both genotypes were smooth. Wild-type anthers developed into tetrads at stage 8b and then normally released microspores at stage 9 (Fig. 3M–O, m–o), whereas in *osabcg15*, the bound microspores in the tetrads at stage 8b and the free microspores at stage 9 appeared wizened more frequently than in wild type (Fig. 3N–O, n–o). Some smooth microspores could be found in *osabcg15* (Fig. 3X). The *osabcg15* microspores began to degenerate by stage 10 and no microspores except for residues could be seen in subsequent stages because of complete degeneration (Fig. 3p–r). Wild-type anthers were filled with enlarged and round mature pollen with elaborate exine patterning (Fig. 3P–R).

**Fig. 4:**
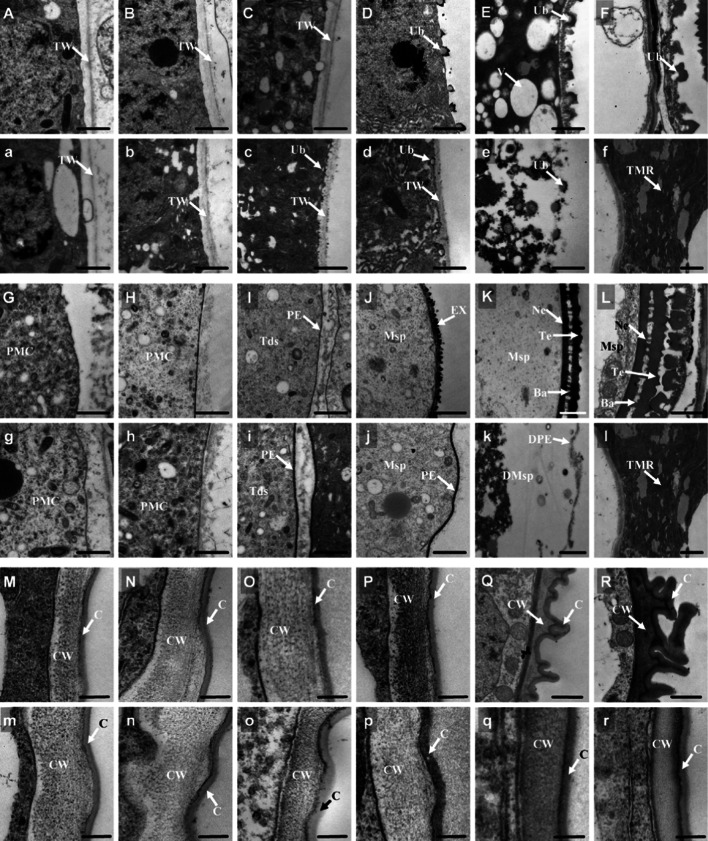
Comparison of anther and pollen between the wild type and *osabcg15* by transmission electron microscopy. *Ba* bacula, *C* cuticle, *CW* cell wall, *DMsp* degenerated microspore, *DPE* degenerated prim-exine, *EX* exine, *Msp* microspore, *Ne* nexine, *PMC* pollen mother cell, *PE* prim-exine, *Te* tectum, *Tds* tetrads, *TMR* tapetum and microspore residue, *TW* tapetum cell wall, *Ub* Ubisch, *V* vacuole. *Bars* 1 μm in **A**–**L**, and **a**–**l**; 200 nm in **M**–**R**, and **m**–**r**. Cross-sections of the wild-type tapetum at stages 7 (**A**), 8a (**B**), 8b (**C**), 9 (**D**), 10 (**E**), and 11 (**F**). Cross-sections of the *osabcg15* tapetum at stages 7 (**a**), 8a (**b**), 8b (**c**), 9 (**d**), 10 (**e**) and 11 (**f**). **G**–**L** The pollen exine development of the wild type from stages 7–11. **g**–**l** Defective pollen exine development of *osabcg15* from stages 7–11. **M**–**R** Outer region of anther epidermis in the wild type from stages 7–11. **m**–**r** Outer region of the anther epidermis in the *osabcg15* mutant from stages 7–11

To improve our understanding about the abnormalities of the internal anther wall and pollen cells in *osabcg15*, TEM was performed. Using the same procedure as previously described using semi-sections of anther, we confirmed the abnormal widening of the middle layer and incomplete degeneration of the endothecium in *osabcg15* (see Supplementary Fig. 1 online). The tapetum and microspores were then compared. In agreement with the semi-section and SEM results, during stages 7–8b, the microspores in *osabcg15* were more frequently wizened than those in the wild type (see Supplementary Fig. 2 online). No significant differences were detected in the tapetum and microspore structures when comparing the wild-type and *osabcg15* (Fig. 4A–C, G–I, a–c, g–i). At stage 9, numerous prim-Ubisch, intermediate to small chifres, were released onto the peripheral side of the tapetum in wild type, indicating the initiation of the secretion of sporopollenin precursor from the sporophytic tapetum (Fig. 4D). Meanwhile, the wild-type microspores formed a primary exine structure, composed of the nexine and baculum, and had a round shape (Fig. 4J). In contrast, the *osabcg15* Ubisch bodies were smaller and failed to break through the tapetal cell wall even though they were created in stage 8b (Fig. 4c, d). At the same time, the exine of *osabcg15* microspores failed to initiate deposition of the sporopollenin-composing materials, and the surfaces of some microspores looked wavy (Fig. 4j; see Supplementary Fig. 2 online). At stage 10 in wild type, the tapetum became condensed along with high vacuolization and the continuation of degradation, while the Ubisch bodies expanded (Fig. 4E). Because of the increased amount of precursors depositing onto the microspore surfaces and then polymerizing, the microspores formed a regular exine structure with nexine, baculum, and tectum on the surface (Fig. 4K). However, the whole tapetum of *osabcg15*, including the peripheral region, dispersed completely, and the immature Ubisch bodies beneath the tapetum cell wall were destroyed (Fig. 4e), indicating serious defects in the synthesis and transport of sporopollenin precursors. Instead of developing into the classical exine bilayer, the thin primexine degraded from a lack of sporopollenin components, and the microspores eventually collapsed (Fig. 4k). In the final stages, except for stage 11, degeneration continued, and the tapetum of wild type was left with just a thin layer of peripheral region to hold the mature Ubisch bodies (Fig. 4F). The microspore exine thickened from the abundant deposition of sporopollenin (Fig. 4L). However, during these stages in *osabcg15*, there was total degeneration of the tapetum and microspores, and no clear structures of tapetum, Ubisch bodies, or microspores could be found in the shriveled locules except for residues produced by the degraded tapetum and microspores (Fig. 4f, l).

To confirm the defects in the anther cuticle found by SEM, we additionally examined the formation of wax crystals. From stages 7–9, no obvious differences could be seen between the wild type and *osabcg15.* Both anther epidermal layers developed a thin layer of cuticle with uniform thickness on the exo-surface. By stage 10, although the width of the cuticle remained unchanged, the wild-type cuticles started to wave and fold and finally formed into a thick curly hair-like structure on the anther epidermis at stage 11. The cell wall of the epidermis thinned increasingly from stage 10 to the last stage (Fig. 4Q, R; see Supplementary Fig. 2 online). In contrast, the *osabcg15* anther cuticle failed to wave and fold, and the cell wall width changed little (Fig. 4q, r; see Supplementary Fig. 2 online). The abnormal development of the cuticle in the *osabcg15* anther epidermis was responsible for the smooth exo-surface seen in the SEM results (Fig. 3c–f).

### Map-based cloning and functional complementation of *OsABCG15*

**Fig. 5:**
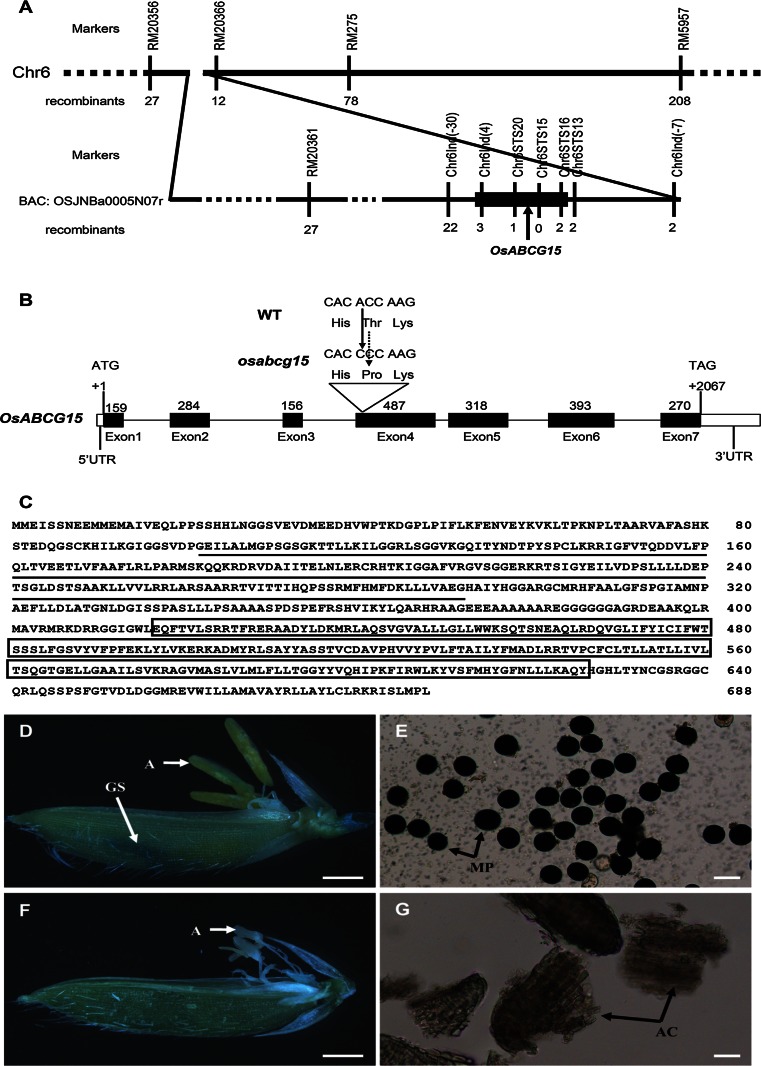
Molecular identification, sequence analysis and functional complementation of *OsABCG15.* **a** Fine mapping of the *OsABCG15* gene on chromosome 6. Marker names and recombinant numbers are indicated on the *vertical line*. The point mutation was limited to a 2.2-kb region between molecular markers Chr6STS20 and Chr6STS16 within the target gene (*black box*). **b** Schematic representation of the exon and intron organization of *OsABCG15*. The mutant sequence has an A-to-C transition in the fourth exon. +1 indicates the translation start site, with the stop codon (TAG) at +2067. *Black boxes* indicate exons; *intervening lines* indicate introns; *white boxes* indicate untranslated regions. **c** Protein sequence of *OsABCG15*. The predicted ATP-binding and ABC-2 transmembrane domains are indicated by an *underline* and a *white box*, respectively. GUS-stained flowers and I_2_–KI stained pollen from the complemented line (**d**, **e**) and the *osabcg15* mutant (**f**, **g**). *A* anther, *AC* anther chips, *GS* GUS stained spot, *MP* mature pollen. *Bars* 1 mm in **d** and **f** and 50 μm in **e** and **g**

The target gene was previously located between the SSR markers RM7434 and RM275 on chromosome 6 (Zhang et al. 2008b). To localize the mutant gene more precisely, 2,157 sterile individuals from an F_2_ mapping population were analyzed using a set of linked primer pairs (see Supplementary Table 1 online). This sterile gene was located between two markers, Chr6STS20 and Chr6STS16, and these two markers were located in the BAC clone of OSJNBa0005N07r. No recombinant was found after detection using another close marker, Chr6STS15, seated between Chr6STS20 and Chr6STS16 (Fig. 5a). Fortunately, we localized the target gene quite precisely, such that the defined region only covered about 2.2 kb of DNA. Single and double recombinants were detected using the gene-riding markers Chr6STS20 and Chr6STS16, respectively. Because this defined region was contained in an ABC transporter gene LOC_Os06g40550 (*OsABCG15*), *OsABCG15* was identified as the candidate target gene. Via PCR amplification and sequencing, we found an A-to-C transition in the fourth exon that caused an amino acid substitution of Thr-to-Pro in the predicted ATP-binding domain of the protein sequence (Fig. 5b). The threonine was conserved among 25 orthologs from 12 species (Supplementary Table 2), indicating that the Thr-to-Pro substitution might be responsible for *OsABCG15* loss-of-function in the *osabcg15* mutant.

In public databases such as NCBI, RGAP, and Gramene, *OsABCG15* is proposed to contain seven exons and six introns. To verify this gene structure, we determined the mRNA splicing pattern by sequencing the cDNA fragment amplified by RT-PCR. A comparison with the genomic sequence confirmed the predicted numbers of exons and introns. *OsABCG15* putatively encodes an ABC transporter protein containing an ATP-binding domain of 191 amino acids in the N-terminal region and an ABC-2 transmembrane domain of 210 amino acids in the C-terminal region (Fig. 5c).

To confirm that the mutation in this ABC transporter gene was responsible for the sterility observed in *osabcg15*, an 8,543-bp genomic DNA fragment containing the entire *OsABCG15* coding region was digested from a BAC clone (OSJNBa0005N07r) and linked to *pCAMBIA1301* carrying a GUS reporter to generate the *p1301OsABCG15* construct. This binary plasmid was then transformed into the homozygous *osabcg15* mutant. Normal anthers and pollen developed in these transgenic plants in the T_0_ generation (Fig. 5d, e), and the restoration of fertility was co-separated with GUS coloration in the T_1_ generation (see Supplementary Fig. 3 online). These results demonstrated that the Thr-to-Pro substitution in *OsABCG15* was responsible for the developmental defects in this mutant.

### OsABCG15 belongs to a subfamily of ABC transporters conserved among terrestrial plants and is a plasma-membrane localized protein

There are about 130 ABC transporters in rice; 50 of them belong to the ABCG subfamily, which has two subgroups: WBC (half-sized, 30 members) and PDR (pleiotropic drug resistance, full-length, 20 members) (Verrier et al. 2008). As a typical member of the WBC subgroup, *OsABCG15* encodes a half-sized ABC transporter of 688 amino acids with one NBD (underlined in Fig. 5c) and one TMD (boxed in Fig. 5c).

To gain additional insight from the phylogenetic relationships among *OsABCG15* and its close homologs, we searched public databases, mainly NCBI using BLASTP, with the amino acid sequence as a query and retrieved 25 closely related homologs. Including OsABCG15, these 26 proteins existed in 12 different species of mosses, pteridophytes, and angiosperms (see Supplementary Table 2 online). Sequence comparison (see Supplementary Fig. 4 online) indicated that all 25 proteins had highly similar NBD and TMD domains, implying that these two functional domains are evolutionarily conserved from mosses to angiosperms.

**Fig. 6:**
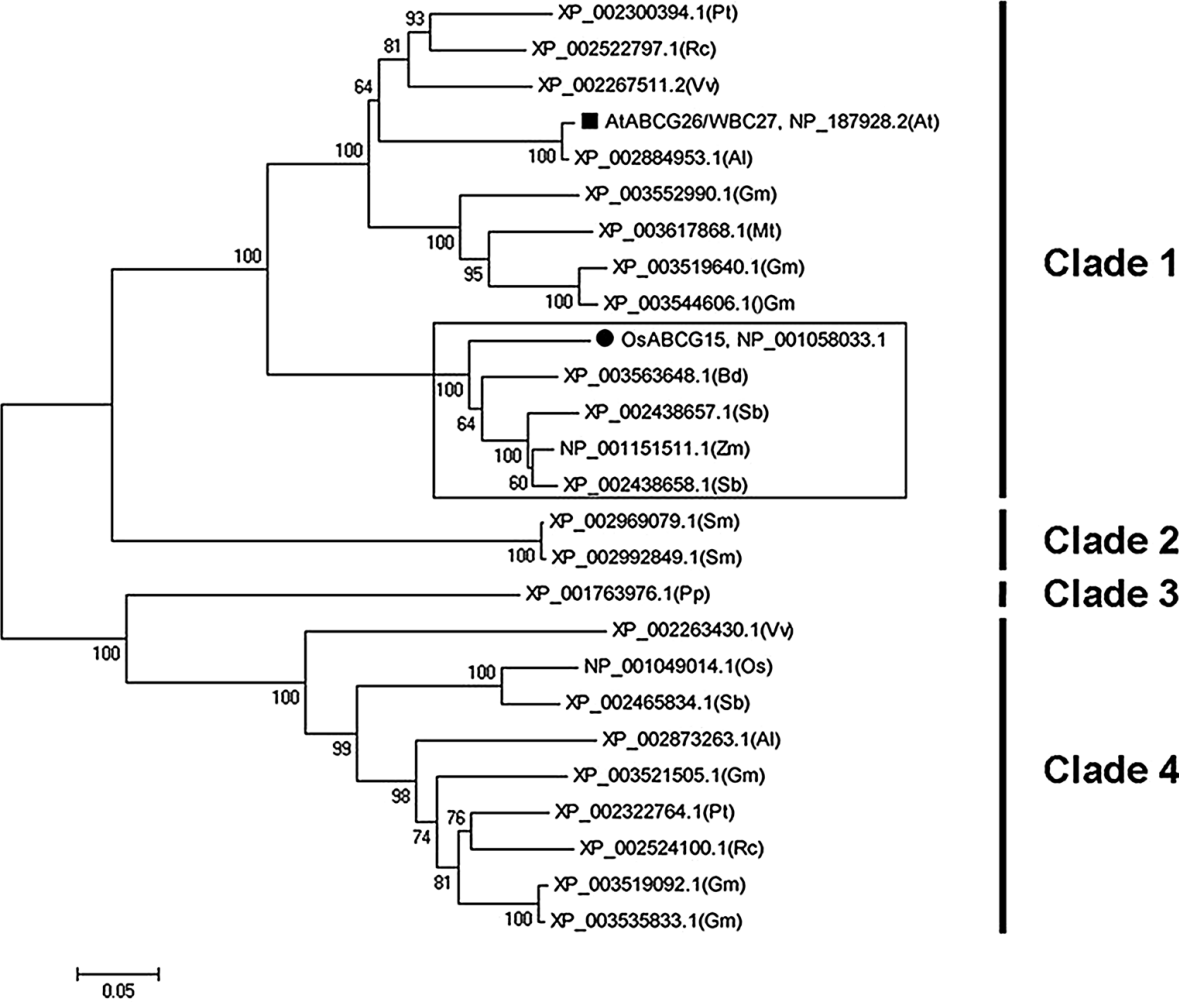
Phylogenetic analysis of OsABCG15 and related proteins. A bootstrap neighbor-joining phylogenetic tree was constructed using MEGA 5.0 and 1,000 replicates. The *number* on each *interior branch* is the bootstrap percentage. The *scale bar* indicates the estimated number of amino acid substitutions per site (for alignment, see Supplementary Fig. 4 online) and the proteins are named by their NCBI accession numbers. *Black dots* and *squares* indicate OsABCG15 and AtABCG26/WBC27, respectively. At *Arabidopsis* *thaliana*; Al *Arabidopsis lyrata*; Bd *Brachypodium distachyon*; Gm *Glycine max*; Mt *Medicago truncatula*; Os rice; Pp *Physcomitrella patens*; Pt *Populus trichocarpa*; Rc *Ricinus communis*; Sb *Sorghum bicolor*; Vv *Vitis vinifera*; Zm *Zea mays*

Subsequently, a neighbor-joining phylogenetic tree of the 26 genes was constructed (Fig. 6). It showed that OsABCG15 was clustered in a Poaceae sub-clade (boxed in Fig. 6) together with four other proteins from *Brachypodium distachyon*, *Sorghum bicolor* and *Zea mays*. In this sub-clade, OsABCG15 homologs were encoded by a single subfamily member in rice, *Brachypodium distachyon,* and *Zea mays*, but not *Sorghum bicolor*. Among the species sampled, only *Medicago truncatula*, *Brachypodium distachyon* and *Physcomitrella patens* contained a single copy of the gene, while two or more orthologs existed in other species. For example, there were six different loci encoding this protein in *Glycine max*. Remarkably, clade 1 and clade 4 diverged quite early, although both comprised similar homologues from angiosperms. After searching the eFP (electronic fluorescent pictograph) browser in the Bio-Array Resource (BAR, http://bar.utoronto.ca/welcome.htm), we found that most of the homologs in clade 1 were expressed mainly in flowers, and most in clade 4 were predominantly expressed in leaves. Thus, these ABC transporter members have evolved different biological functions, although all have conserved functional NBD and TMD domains.

**Fig. 7:**
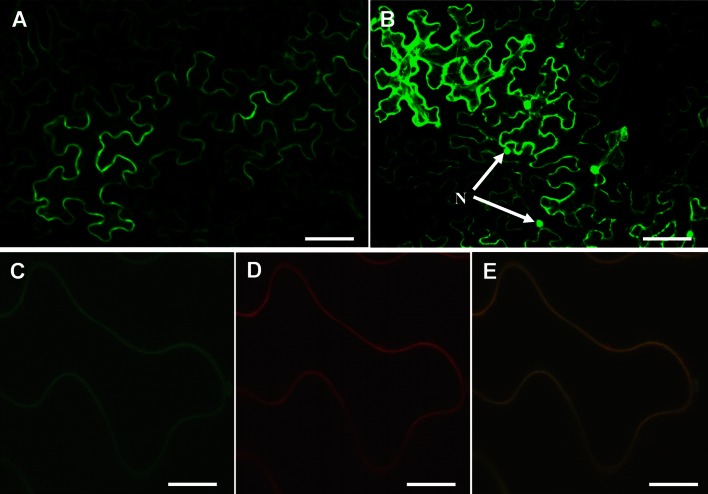
Subcellular localization of the GFP-OsABCG15 fusion protein in *Nicotiana benthamiana* epidermal cells. **a** Transient expression of the GFP-OsABCG15 fusion protein, showing localization of the GFP-OsABCG15 protein on the plasma membrane. **b** Transient expression of the 35S:GFP control protein, showing that the expression of the GFP protein was distributed throughout the cell, including the cytoplasm and nucleus. **c** GFP-OsABCG15 imaging. **d** Fluorescent signal image of a FM4-64 stain. **e** Merged image of **c** and **d**. *Scale bars* 100 μm (**a**, **b**) and 10 μm (**c**–**e**)

AtABCG26/WBC27, an *Arabidopsis* *thaliana* homolog of OsABCG15, was located in a separate dicot sub-clade of the phylogeny. Recent reports indicate that AtABCG26/WBC27 plays an important role in exporting sporopollenin precursors from tapetal cells and is a plasma membrane protein (Quilichini et al. 2010; Dou et al. 2011; Choi et al. 2011). In light of the homology of these two proteins and the similar pollen development defects in *atabcg26/wbc27* and *osabcg15* mutants, we predicted that OsABCG15 is also localized to the plasma membrane. To confirm this, we fused the full-length *OsABCG15* coding region with *GFP* using a *GFP*-*OsABCG15* pattern to screen recombinants. The fused construct and the *GFP*-only control, both driven by the cauliflower mosaic virus 35S promoter, were introduced into *Nicotiana benthamiana* epidermal cells by *Agrobacterium* infiltration (Voinnet et al. 2003). As expected, the GFP-OsABCG15 fusion protein was observed exclusively on the plasma membrane (Fig. 7a, c). In contrast, the free GFP signal occurred throughout the cell (Fig. 7b). Moreover, the green fluorescence, observed on the periphery of the *GFP*-*OsABCG15* transformed cells, was similar to a membrane-specific FM4-64 stain signal (Bolte et al. 2004), and the two signals overlapped on the plasma membrane after merging the photomicrographs from each gene product (Fig. 7c–e). These observations support the prediction that OsABCG15 is localized to the plasma membrane and are consistent with the presumed function of exporting lipid-soluble precursors across the tapetal plasma membrane, resulting in the subcellular location observed in AtABCG26/WBC27.

### *OsABCG15* is preferentially expressed in the tapetum during anther development

**Fig. 8:**
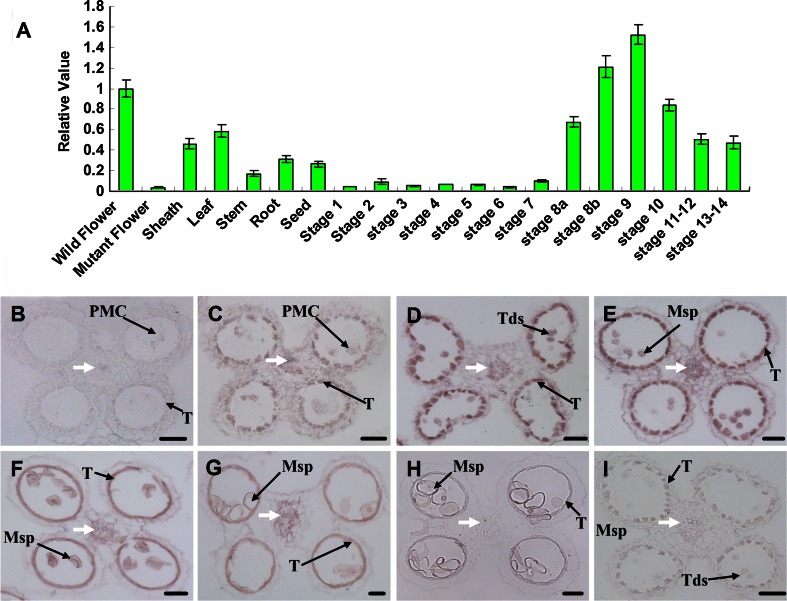
Expression pattern of *OsABCG15.* **a** Expression analysis of *OsABCG15* by RT-qPCR. The wild-type and mutant samples were mixed from their respective flowers from stages 8–11 in triplicate. RNA in situ analysis of *OsABCG15* in wild-type anthers. **b** Stage 7. No expression. **c** Stage 8a. Hybridization signal localized to the tapetum. **d** Stage 8b. Enhanced expression in the tapetum. **e** Stage 9. Peak expression in the tapetum. **f** Early Stage 10. High expression in the tapetum. **g** Stage 10. Reduced expression in the tapetum. **h** Stage 11. No visible signal in the tapetum. **i** Stage 8b. No visible signal in the wild-type anther with the sense probe. *White arrows* indicate vascular connective tissue. *PMC* pollen mother cell, *Msp* microspore, *T* tapetum, *Tds* tetrads. *Bars* 25 μm

Defects in the *osabcg15* mutant occurred only in reproductive organ of the anther, with no obvious abnormal phenotypes in vegetative organs, indicating that *OsABCG15* might be preferentially expressed in the anther. To evaluate the expression pattern of *OsABCG15*, we queried the BAR rice eFP browser (http://www.bar.utoronto.ca/efprice/cgi-bin/efpWeb.cgi). The results suggested that *OsABCG15* expression increases substantially in a 3- to 10-cm inflorescences and peaks in a 10- to 15-cm inflorescences, after which it decreases in mature anthers. Compared to the floral expression, *OsABCG15* expression in vegetative organs was reduced. To confirm these spatial and temporal expression patterns, quantitative RT-qPCR of rice various organs, including flowers at different stages, was conducted (Fig. 8a). The results indicated that *OsABCG15* was strongly expressed in the middle and late floral stages, beginning at stages 7–8a, increasing and peaking at stages 8b–9, and finally declining at stages 10–14. In contrast, very little expression was detected in vegetative organs. Meanwhile, RT-qPCR revealed weak transcription levels in mutant flowers, suggesting that *OsABCG15* was completely downregulated in *osabcg15* flowers.

To determine the precise tissue specificity of *OsABCG15* expression in anthers, we performed RNA in situ hybridization with wild-type anther sections. At stage 7, no hybridization signal was detected (Fig. 8b). Expression of *OsABCG15* was visible in the tapetum at stage 8a (Fig. 8c). Maximal expression was observed in the tapetum at stages 8b and 9, when the tetrads were produced and young microspores released (Fig. 8d, e). At stage 10, when vacuolated pollen formed, the expression began to decline (Fig. 8f, g). Finally, the expression of *OsABCG15* was invisible at stage 11, when the tapetum degenerated completely and lentiform microspores appeared (Fig. 8h). From stages 8b to 10, we observed notable signals in the vascular regions of connective tissue and in early microspores (Fig. 8d–g). No signal was detected in other anther tissues, such as epidermis and endothecium, at any stage. Notably, no positive signal other than background was observed through the sense probe at any stages (Fig. 8i; see Supplementary Fig. 5 online). Based on these results and those of RT-qPCR, we concluded that *OsABCG15* is preferentially expressed in the anther and is strongly transcribed in the tapetum at stages 8b–10. These *OsABCG15* expression patterns were consistent with the data from the BAR rice eFP browser and are similar to the Arabidopsis ABCG26/WBC27 expression patterns recently reported (Choi et al. 2011; Dou et al. 2011; Quilichini et al. 2010). They also agreed with the abnormal phenotypes noted in the anther and pollen development of *osabcg15*.

## Discussion

### *OsABCG15* is essential for normal development of the anther cuticle and pollen exine

To protect microspore/pollen grain from environmental and biological stresses, plants have evolved two elaborate lipid-soluble protective barriers, the anther cuticle and the pollen exine. This study identified the function of *OsABCG15*, an ABC transporter gene. A mutation in *OsABCG15* led to obvious and drastic defects in Ubisch body formation, tapetal and middle-layer degeneration, and anther cuticle and pollen exine development in rice.

The first notable defect due to the functional loss of *OsABCG15* in the mutant was in Ubisch body generation. Compared with the numerous Ubisch bodies extruding through the tapetum cell membrane at stage 9 in the wild type, fewer and more abnormally sized Ubisch bodies formed in the mutant, and none could transfer from the peritapetal region. These prim-Ubisch bodies were removed with the abnormal degradation of the tapetum.

Another significant difference was found in the degeneration of the tapetum and middle layer. In wild type at stage 10, the peritapetal region was still present with numerous Ubisch bodies, although the tapetum degenerated continually and the cytoplasm condensed. The middle layer became almost invisible. However, the degeneration of tapetum in the *osabcg15* mutant was abnormal because the whole tapetum, including the peritapetal region, fell apart and dispersed, and the middle layer were abnormally enlarged. Because numerous Ubisch bodies remained in the peritapetal region, we believe that the normal tapetum degeneration of wild type in the final stages is partial rather than complete, as was previously thought. The inclusion-exhausted tapetum appears to be dissipated because it is pressed into a thin, nearly invisible layer by the copious enriched pollen. Subsequently, the microspore exine, the cuticle of the anther epidermal cells, and the connective tissue cells, including stomata, failed to initiate formation because of the lipid-deposition defects. The released microspores soon degenerated completely as a result of the loss of these two protective barriers.

These defects blocked the normal development of pollen and resulted in complete male sterility in *osabcg15*. Mutant sterility could be restored in a genetic complementation test, indicating that the *OsABCG15* gene was responsible for the normal development of rice pollen.

Three reports (Qin et al. 2013; Zhu et al. 2013; Niu et al. 2013) described defects in different mutants of *OsABCG15* at the time of our manuscript submission. The characterizations of these *osabcg15* mutants reflected our findings regarding the undeveloped or absent Ubisch bodies, abnormal degradation of the tapetum, unformed exine of the microspore and cuticle of the anther epidermis, and the complete degeneration of the microspore. The abnormally expanded middle layer described in this study was also observed by Zhu’s group but was not noted in the other reports (Qin et al. 2013; Zhu et al. 2013; Niu et al. 2013). Overall, these studies suggested that *OsABCG15* plays a crucial role in lipid deposition in the anther cuticle and pollen exine in rice, even though some other defects in *osabcg15* mutants were described in these reports. In our study, we initially noted successive and centrifugal growth of wax on the wild-type anther epidermis, which provided valuable information regarding the underlying mechanism of cuticle formation.

Prior to this study, three parallel reports on Arabidopsis showed that *WBC27*/*ABCG26*, the homolog of *OsABCG15*, plays a similar role in pollen exine development (Quilichini et al. 2010; Choi et al. 2011; Dou et al. 2011). Using the same T-DNA insertion mutant in SALK_062317, researchers discovered a failure to deposit lipids in the pollen exine, causing a lack of exine formation in the microspores and ultimately leading to the degeneration of microspores once released from their tetrads. Only a few pollen grains survived to pollinate of the stigmas and allow some seed production, despite harboring this complete gene knockout. In fact, the Arabidopsis anther cuticle had a longitudinal striped pattern (Zhang et al. 2011). Unfortunately, those observations in Arabidopsis did not reveal whether or not the outer barrier, the anther cuticle, was abnormal in the mutants. Although this mutation’s effects on the Arabidopsis anther cuticle were undefined and the resulting male infertility was not as complete as in rice, the two homologs of ABC transporter genes are genetically conserved in exine lipid deposition.

### The role of *OsABCG15* in lipid-soluble material deposition in the anther cuticle and pollen exine

Cutin, wax, and sporopollenin synthesis require movement of their secreted precursors to the epidermis and microspore. These actions involve at least three processes: (1) de novo synthesis of precursors, (2) massive secretion from the lipid bilayer to the apoplastic compartment, and (3) transfer/translocation of the precursors to their target surfaces. Several genes have been identified to be involved in the biosynthesis of cuticle and exine components, such as *MS2* (Aarts et al. 1997), *CYP703A2* (Morant et al. 2007), *NEF1* (Ariizumi et al. 2004), and *RPG1* (Guan et al. 2008) in Arabidopsis, and *TDR* (Li et al. 2006; Zhang et al. 2008a), *WDA1* (Jung et al. 2006), *CYP703A3* (Aya et al. 2009), *CYP704B2* (Li et al. 2010), and *DPW* (Shi et al. 2011) in rice. Recent studies have shown that the LTPs, such as FLP1 (Ariizumi et al. 2003), type I LTP (At3g51590), and type II LTP (At1g66850) in Arabidopsis and OsC6 in rice, are involved in transporting lipid-soluble precursors during exine formation (Xu et al. 2010; Zhang et al. 2010). An ABC transporter of AtABCG26/WBC27 in Arabidopsis was reported by three research groups to be related to sporopollenin precursor secretion (Choi et al. 2011; Dou et al. 2011; Quilichini et al. 2010).

The ABCG/WBC subfamily is the largest of the plant ABC transporter sub-families. ABCG transporters have a possible role in the export of wax and cutin monomers. Most of the known plant *ABCG* genes are required for the extracellular secretion of lipid-soluble precursors from the epidermis and/or tapetal cells. Functional losses of these lipid-transporting genes always result in defects in epicuticle and/or exine formation. For example, AtABCG11 transports long- and very-long-chain fatty acids, which are precursors of cutin and wax, respectively. Knockout of *AtABCG11* decreases the wax load in stems and the accumulation of intracellular lipid-soluble inclusions (Bird et al. 2007).

OsABCG15 belongs to the ABCG/WBC subfamily. Expression of *OsABCG15* was mainly detected in tapetal cells from stages 8–10 of anther development. The OsABCG15 protein localized to the plasma membrane. A mutation in *OsABCG15* resulted in serious defects in lipid material deposition onto the surfaces of the anther epidermis and microspores. The mutant, *osabcg15*, had a smooth anther epidermis and pollen surface due to a lack of cuticle and exine, respectively. Because OsABCG15 belongs to the same subfamily of ABC transporters as AtABCG11, *OsABCG15* may also be involved in transporting fatty acids or other modified forms. Characterization of the defects in the mutant, the gene expression pattern in the wild type, and the subcellular-location of *OsABCG15* allowed us to hypothesize that *OsABCG15* is a new candidate gene involved in exporting cuticle and exine materials in the rice tapetum, in agreement with the putative transport role of AtWBC27/ABCG26 in Arabidopsis.

### Precursors might be secreted from the tapetum by OsABCG15 and translocated to the epidermis and microspore surface by OsC6

Thus far, most of the lipid-soluble precursor synthesis genes were thought to be expressed mainly in tapetal cells, with the only exception being *WDA1*, implying that the synthesis reaction occurred predominantly in the tapetum. Clearly, the precursors must be secreted from tapetum and transferred onto the surfaces of the anther and pollen walls to polymerize into biopolymers of sporopollenin, wax, and cutin. OsABCG15 and AtWBC27/ABCG26 were thought to be transporters of those precursors in rice and Arabidopsis, respectively. Some, or all, of the products of those lipid-synthesis genes may be exported by OsABCG15 or other transporters. Discovering the exact substrates of OsABCG15 will be interesting. However, the specific constituents of anther cuticle and pollen exine are variable and complicated, as described in the Introduction. The exact biochemical nature of these lipid-soluble precursors remains elusive due to technical limitations, which impede the discovery of all of the lipid-soluble materials carried by OsABCG15. Undoubtedly, identifying the substrates of OsABCG15 will prove to be a difficult yet an attractive challenge for future studies.

Once secreted by OsABCG15 through the tapetal membrane, the precursors should be translocated to the surface of the anther epidermis and microspore to form cuticle and exine. Before this can occur, the potential transporters must cross the hydrophilic tapetal cell wall to arrive at the microspore surface, a distance encompassing three layers of anther before reaching the anther exo-surface. OsABCG15 cannot accomplish this transport because it is restricted to the cyto-membrane. Each plant species has a large number of LTPs that nonspecifically transport lipid molecules with low molecular mass (Carvalho and Gomes 2007; Yeats and Rose 2008). Anther-specific LTPs may participate in the transport of fatty acids and/or other sporopollenin precursors from the tapetum to the microspore during exine deposition (Xue et al. 1994). OsC6 is a distinct LTP in rice that is not classified into the previously identified LTP1 or LTP2 subfamilies found in both dicots and monocots. In vitro, OsC6 proteins have lipid-binding activity and are mainly distributed in the locule, the outer space of epidermal cell wall, as well as the extracellular spaces of the endothecium, epidermis, and middle layer. Therefore, as indicated in the proposed model, the OsC6 protein likely transfers lipid-soluble molecules from metabolically active tapetal cells to other anther cells for Ubisch body, pollen wall, and anther cuticle development (Zhang et al. 2010).

**Fig. 9:**
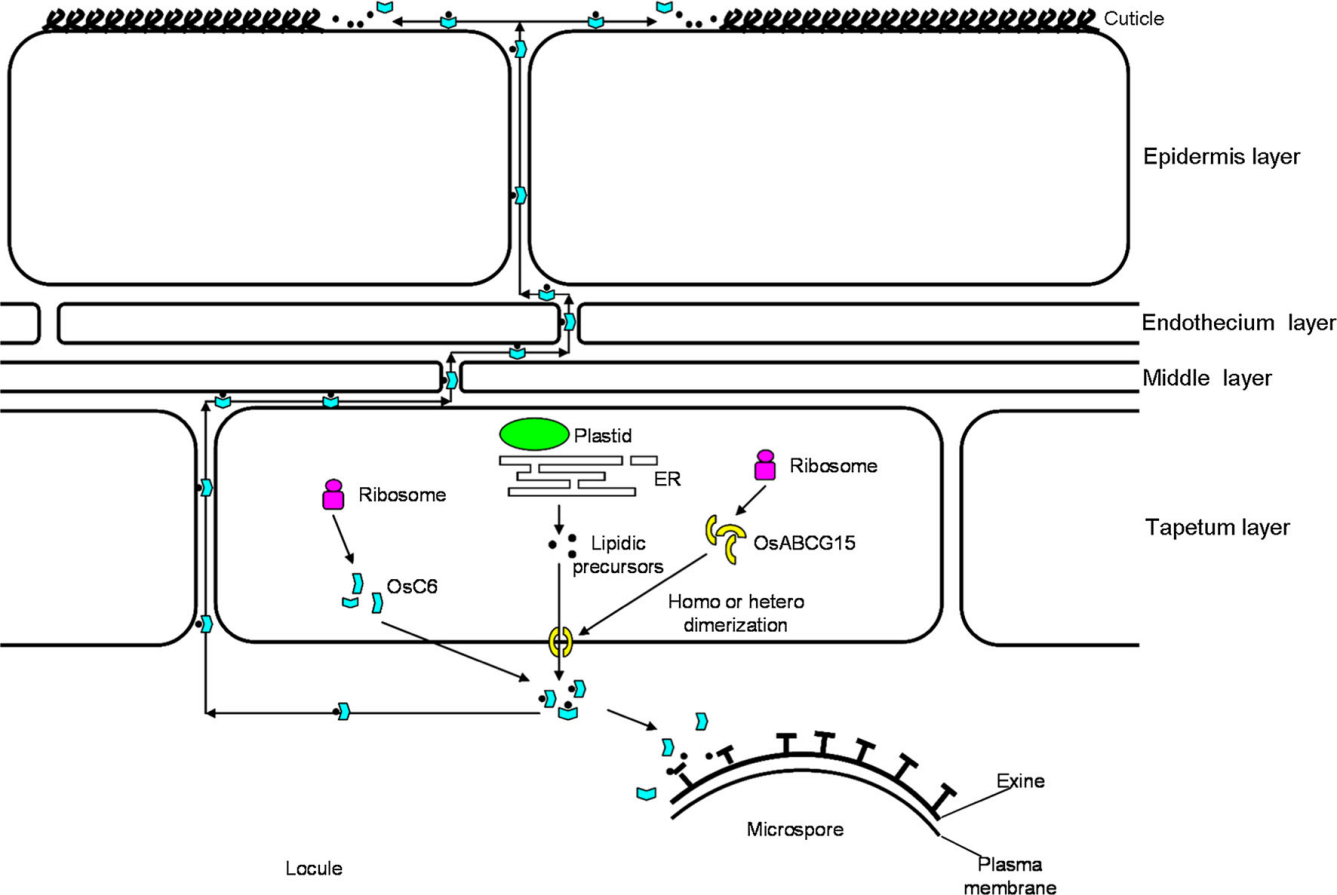
Proposed lipid-soluble precursor transport within the anther. After being translated and modified, OsABCG15s are localized onto the tapetum membrane and are homo/hetero-dimerized. Subsequently, the synthesized lipidic precursors are pumped to the outside of tapetum by the dimerized OsABCG15 proteins. Finally, the secreted OsC6 proteins from tapetum cells carry the lipidic precursors to the surfaces of epidermis and microspores for cuticle and exine development

Two transporters, the ABC transporter OsABCG15 and the LTP OsC6, have been identified for their involvement in lipid-soluble material transportation in anther cuticle and pollen exine formation in rice. Based on our data and recently published models (Zhang et al. 2010; Shi et al. 2011; Choi et al. 2011; Ariizumi and Toriyama 2011) of lipid-soluble precursor synthesis and transport within the anther, we propose a modified model of lipid transport within the rice anther (Fig. 9). In this new model, the lipid precursors are produced first in the tapetum. Subsequently, OsABCG15 forms homo-dimers with itself or hetero-dimers with other half-sized ABCG proteins. The dimerized, full-sized protein acts as a discharge hole on the membrane to secrete the component precursors out of the tapetum. Finally, OsC6 transfers the lipid-soluble precursors to the assembly site (the target surface of anther epidermis and microspore wall) where polymerization occurs.

Although several models of the biosynthesis and transport of lipid-soluble components during anther development have been proposed, many questions remain to be answered. For instance, both *WDA1* and *CYP704B2* are crucial for lipid-soluble material biosynthesis in the normal development of the anther cuticle and pollen exine. A knockout of any of these genes will result in significant and simultaneous defects in cuticle and exine formation. *WDA1* was strongly expressed in the epidermis but only weakly in the tapetum, while *CYP704B2* was expressed only in the tapetal layer and microspores. If the unique spatial expressions of *WDA1* and *CYP704B2* are correct, the different essential precursors related directly to these two genes must be mainly produced in the epidermis and tapetal cells, respectively. Therefore, the precursors from tapetum and epidermis must be different. These findings raise more additional questions: Are the same transporters employed by epidermal and tapetal cells? How are the essential materials from the epidermis secreted and transferred to a different surface? Is there a transport guide to match the different directional flows of the precursors from the epidermal and tapetal cells? Present studies have yet to define the lipid-soluble material transportation mechanism within the anther. To address these questions, more biosynthesis and transport genes must be identified. Now is too early to propose a thorough and exact model of lipid metabolism and transport in anther development, but further analyses will be attempted.

### Accession numbers

Sequence data from this article for the cDNA and genomic DNA of *OsABCG15* can be found in the GenBank/EMBL data libraries under accession numbers NM_001064568 and NC_008399, respectively. Accession numbers for the sequences used in the phylogenetic analysis are on the tree in Fig. 6.

## Electronic supplementary material

Below is the link to the electronic supplementary material.

Supplementary material 1 (TIFF 3779 kb)

Supplementary material 2 (TIFF 3779 kb)

Supplementary material 3 (TIFF 3779 kb)

Supplementary material 4 (TIFF 3790 kb)

Supplementary material 5 (TIFF 3779 kb)

Supplementary material 6 (DOC 8194 kb)

Supplementary material 7 (DOC 38 kb)

Supplementary material 8 (DOC 41 kb)

## Abbreviations

At: *Arabidopsis thaliana*
BAC: Bacterial artificial chromosome
ABC: ATP-binding cassette
GFP: Green fluorescent protein
LTP: Lipid transfer proteins
NBD: Nucleotide binding domain
Os: *Oryza sativa*
PCR: Polymerase chain reaction
SEM: Scanning electron microscopy
SSR: Simple sequence repeats
TEM: Transmission electron microscopy
TMD: Transmembrane domain
VLCFA: Very-long-chain fatty acids
WBC: White–brown complex homolog
