# Supplementary figures and images for: OsABCG15 encodes a membrane protein that plays an important role in anther cuticle and pollen exine formation in rice
Source: Plant Cell Rep. 2014 Aug 20;33(11):1881–99. doi: 10.1007/s00299-014-1666-8 (PMC4197380; doi:10.1007/s00299-014-1666-8)

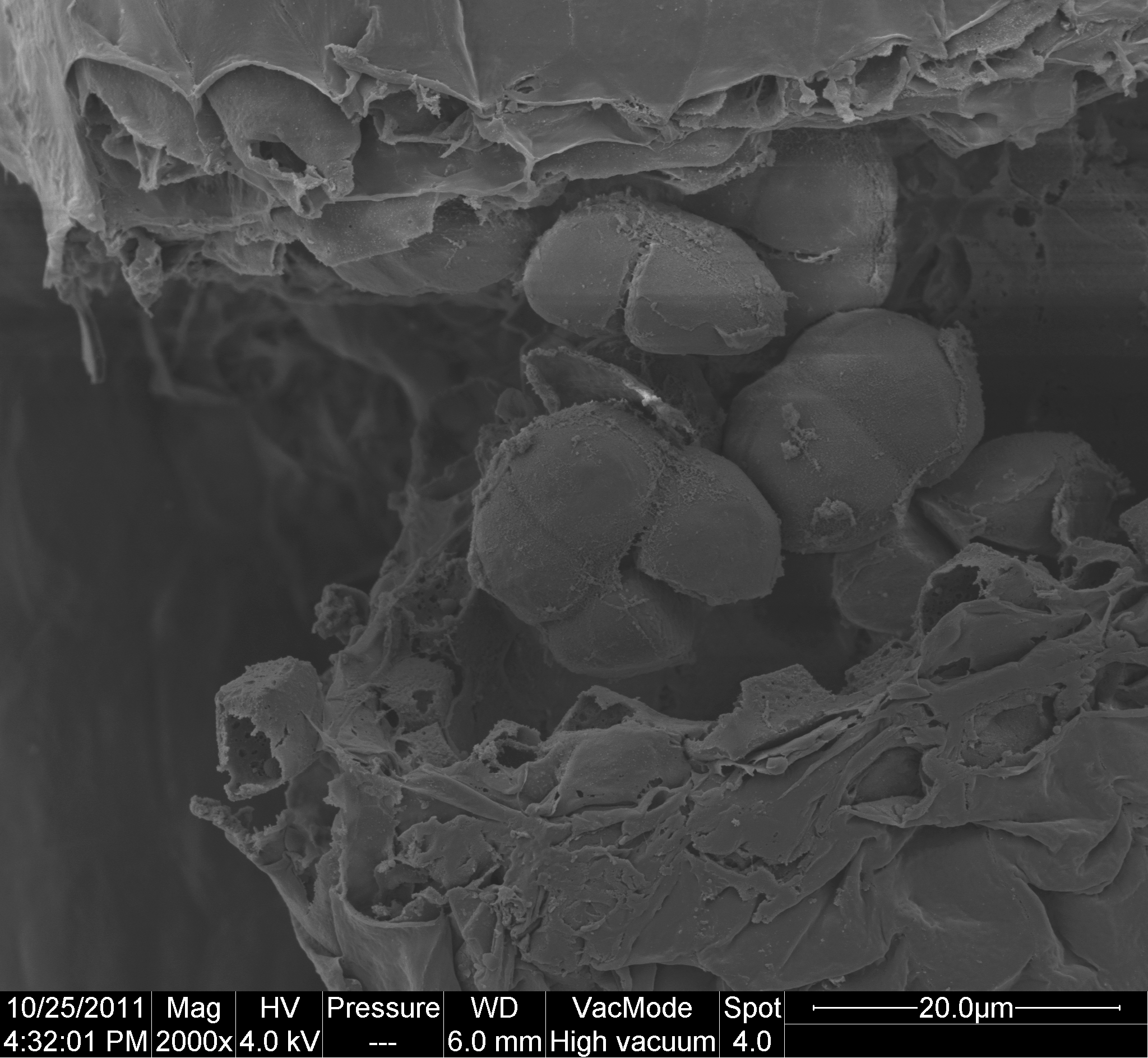

Supplement: Supplementary file 4 — Supplementary material 4 (TIFF 3790 kb) [file 299_2014_1666_MOESM4_ESM.tif]
