# Supplementary material for: OsABCG15 encodes a membrane protein that plays an important role in anther cuticle and pollen exine formation in rice
Source: Plant Cell Rep. 2014 Aug 20;33(11):1881–99. doi: 10.1007/s00299-014-1666-8 (PMC4197380; doi:10.1007/s00299-014-1666-8)
Supplement: Supplementary file 6 — Supplementary material 6 (DOC 8194 kb) [file 299_2014_1666_MOESM6_ESM.doc]

**Supplementary Data**

**
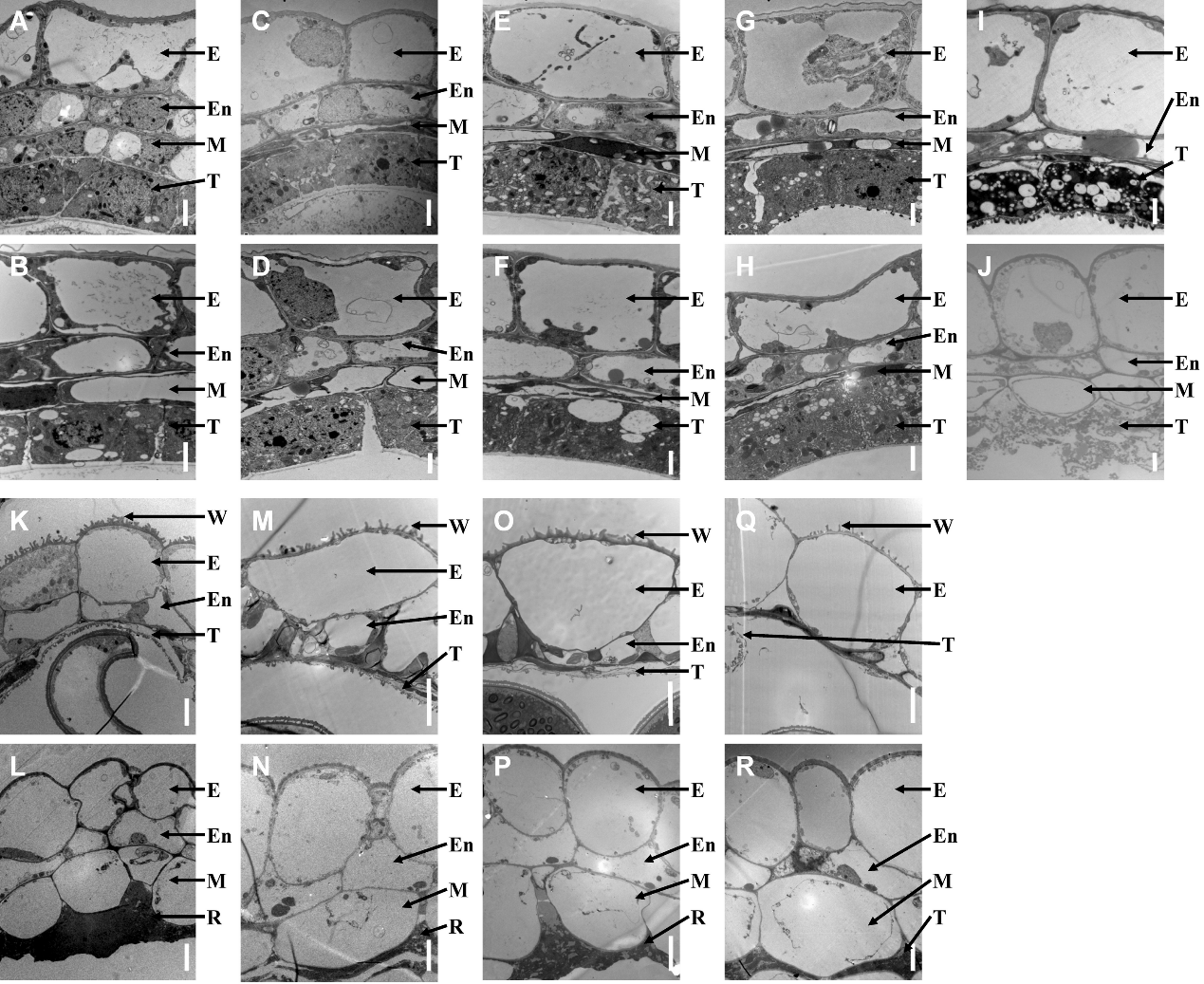
**

**Supplementary Fig. 1** Comparison of the four anther wall layers between the wild-type and *osabcg15*. The upper panels (A, C, E, G, I, K, M, O, and Q) show the four layers of the anther wall sections of wild-type plants, and the lower panels (B, D F, H, J, L, N, P, and R) show the mutant anther sections. E, epidermis; En, endothecium; M, middle layer; R, tapetum and microspore residues; T, tapetum; W, wax crystal. Bars = 2 μm in (A–J); 5 μm in (K–R). (A), (C), (E), and (G) stages 6–9 in wild-type, and (B), (D), (F), and (H) stages 6–9 in *osabcg15*. There were no obvious differences between these wild-type and *osabcg15* tissues*.* (I), (K), (M), (O), and (Q) indicate stages 10–14 in wild-type plants, and (J), (L), (M), (P), and (R) indicate stages 10–14 in *osabcg15* plants. Compared with the gradual disappearance from stage 10 in the wild-type, the middle layer of *osabcg15* is abnormally enlarged, while the endothecal layer was maintained in *osabcg15* and completely degenerated in the wild-type plants

**
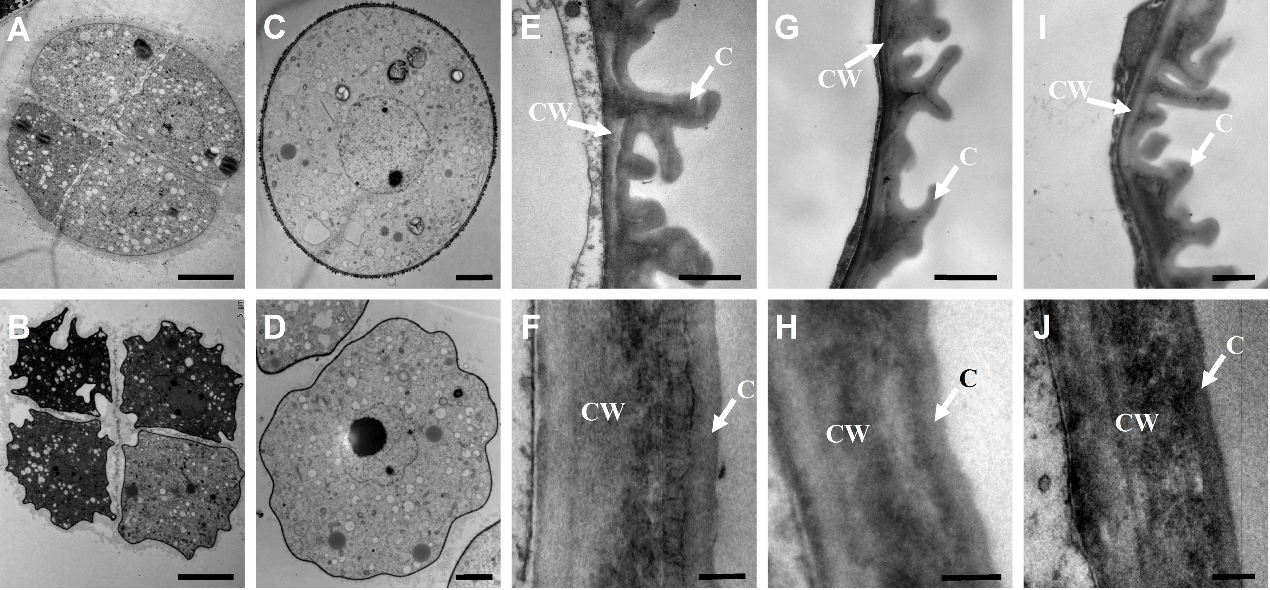
**

**Supplementary Fig. 2** Comparisons of tetrads and microspore shapes, and epidermal cuticles between wild-type and *osabcg15* anthers. C, cuticle; CW, cell wall. Bars = 5 μm in (A) and (B); 2 μm in (C) and (D); 200 nm in (E–J). (A) and (B) Stage 8b in wild-type and *osabcg15* mutants. Microspores are wrinkled in *osabcg15* tetrads, but round in wild-type plants. (C) and (D) Stage 9 in wild-type and *osabcg15* mutants. Microspores are wavy in *osabcg15* locules and round in the wild-type plants. (E), (G), and (I) show stages 12–14 in wild-type, while (F), (H), and (J) show stages 12–14 in *osabcg15* plants. The wild-type epidermal cell wall became progressively thinner, while it remained unchanged in *osabcg15* plants. Also note that the hair-like cuticle failed to form in *osabcg15* plants

**
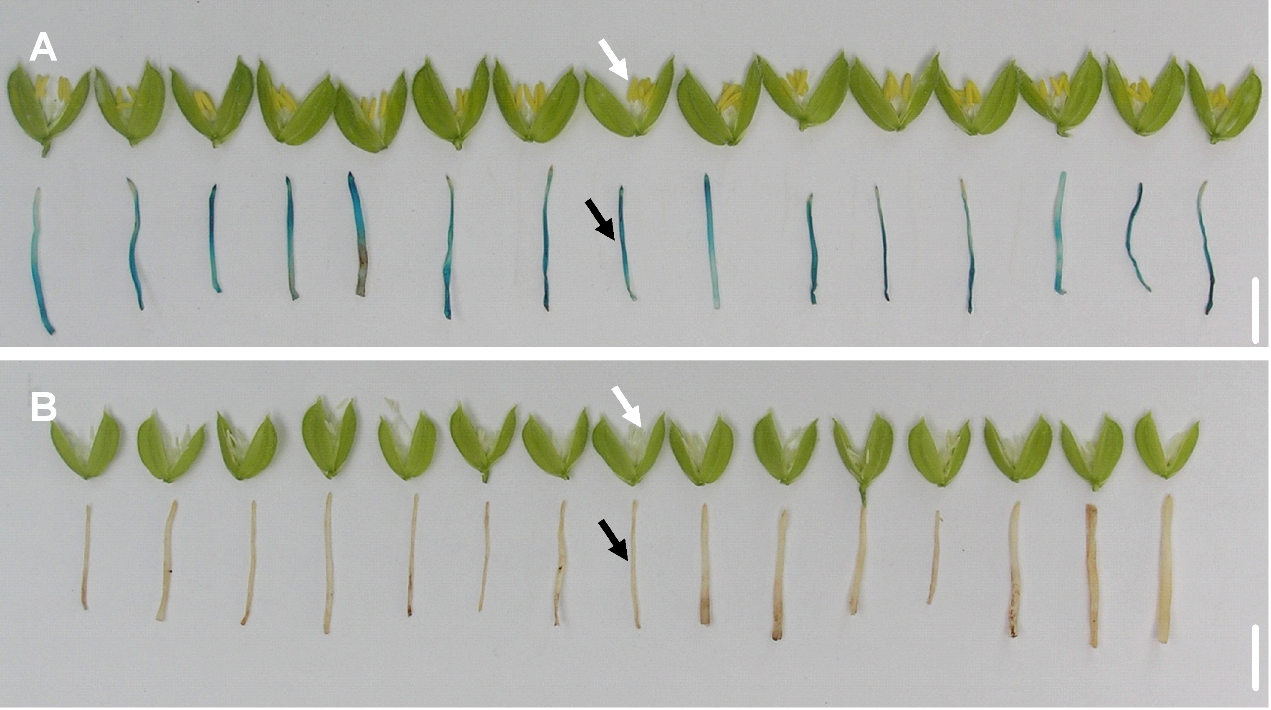
**

**Supplementary Fig. 3** Co-separation of fertility and GUS positive signals in the T_1_ complementation generation. (A) In the T_1_ population, GUS-positive individuals developed yellow fertile anthers. (B) In the T_1_ population, GUS-negative individuals developed white sterile anthers. White arrows indicate anthers and black arrows indicate roots. Bars = 5mm

**
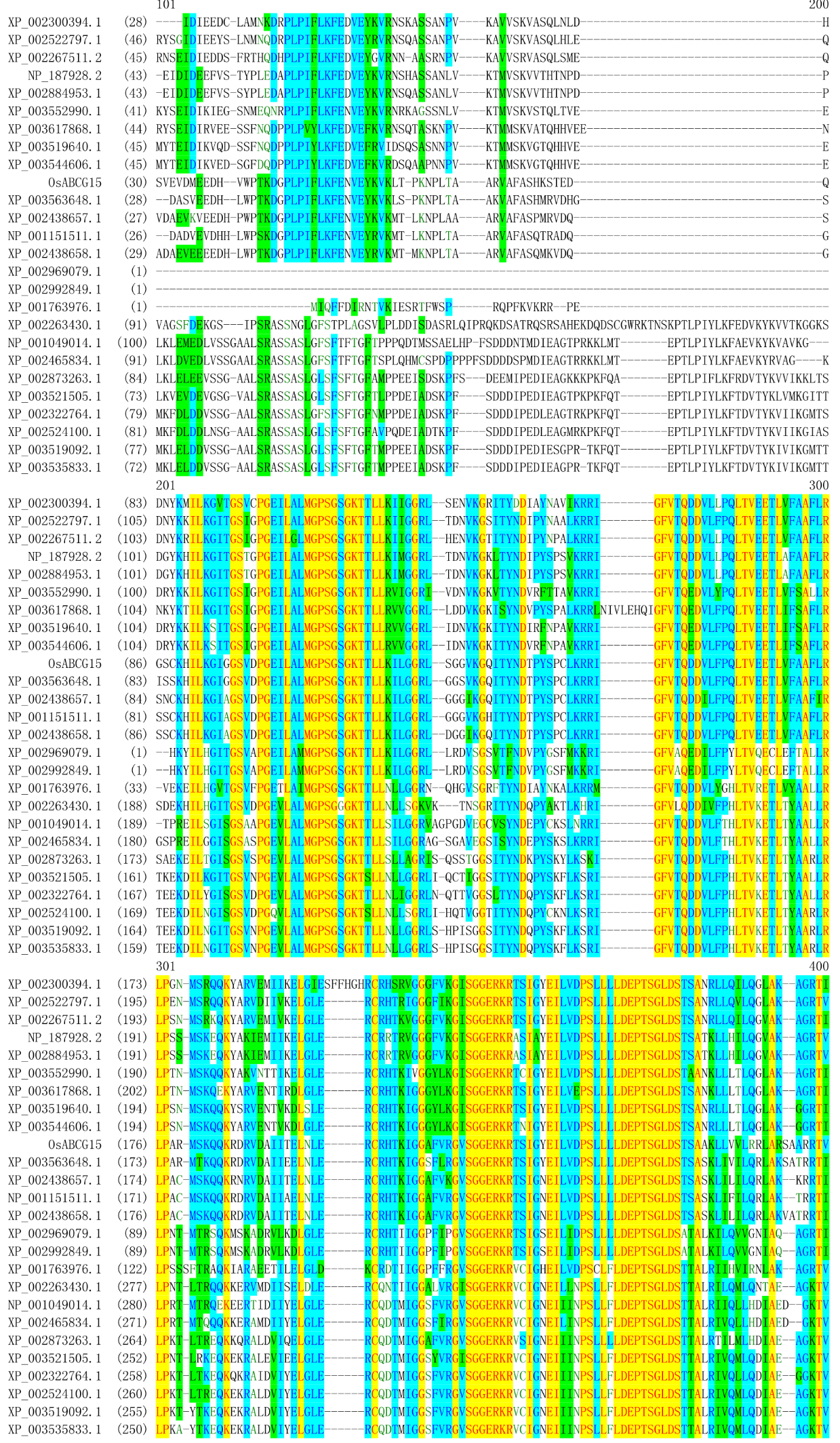

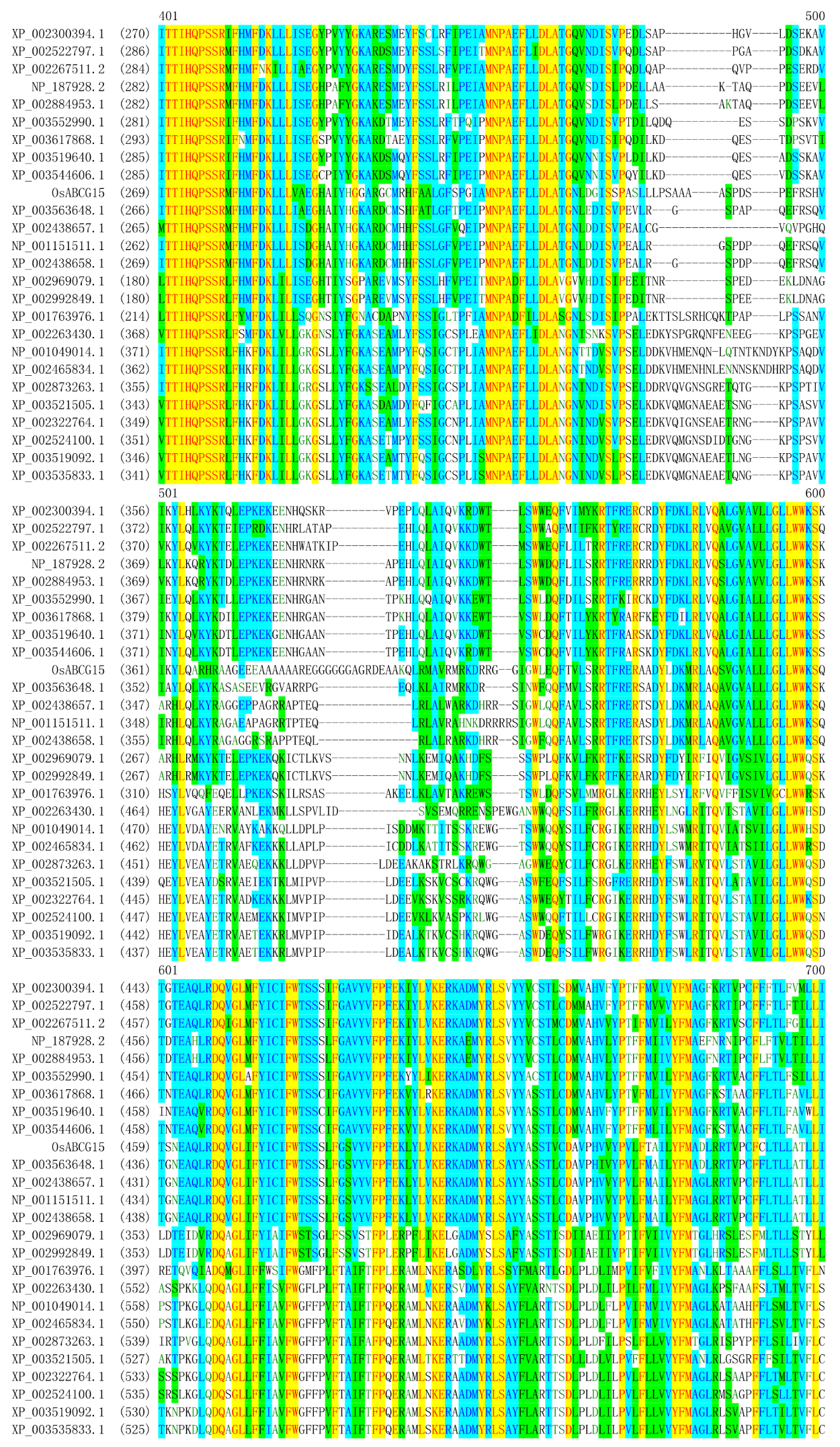

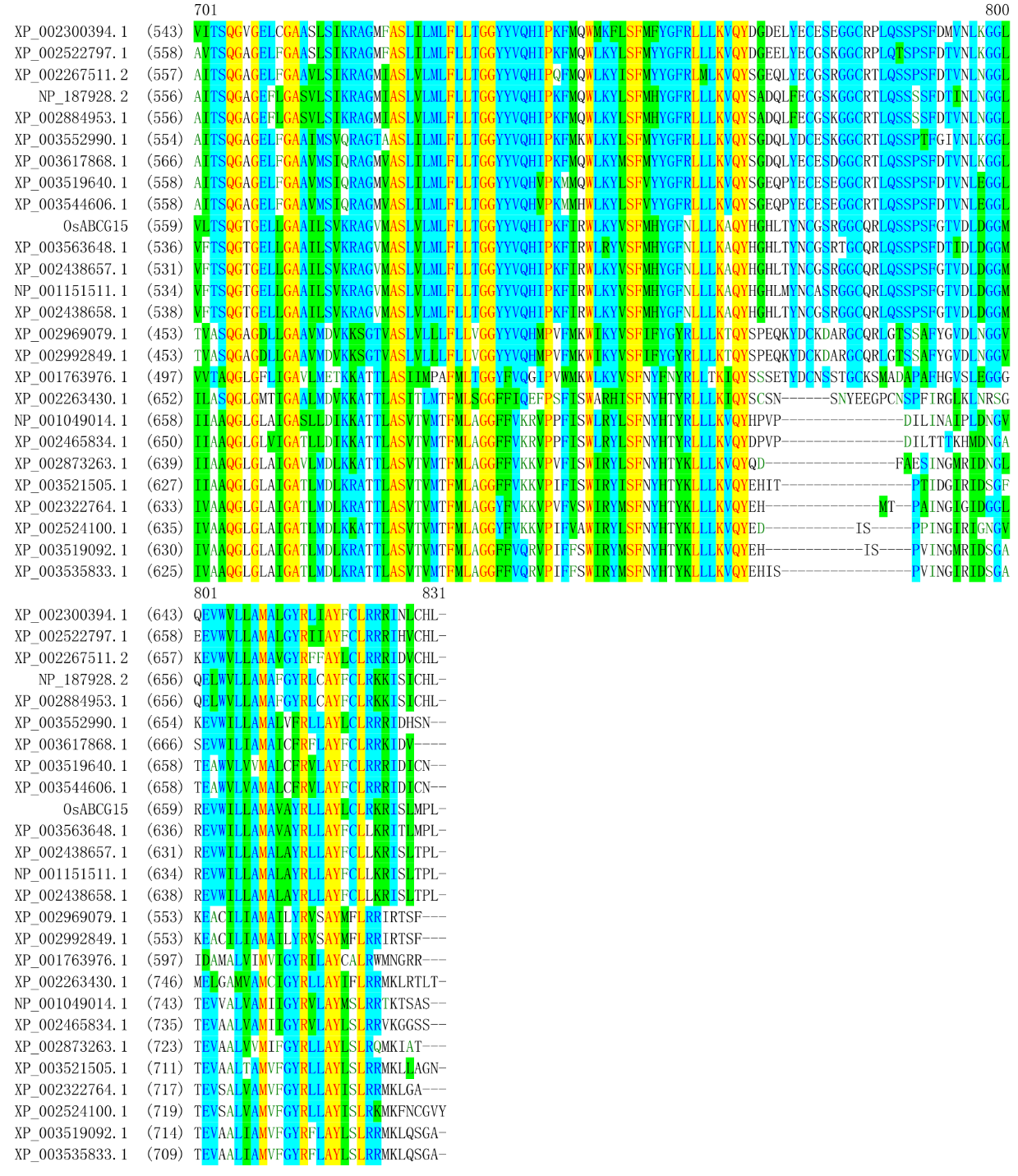
**

**Supplementary Fig. 4** Sequence alignment of OsABCG15 and its close homologs.

Colored boxes indicate the similar amino acid residues. In OsABCG15, amino acids 102–292 are a nucleotide-binding domain (NBD) and 417–626 are a transmembrane domain (TMD)

**
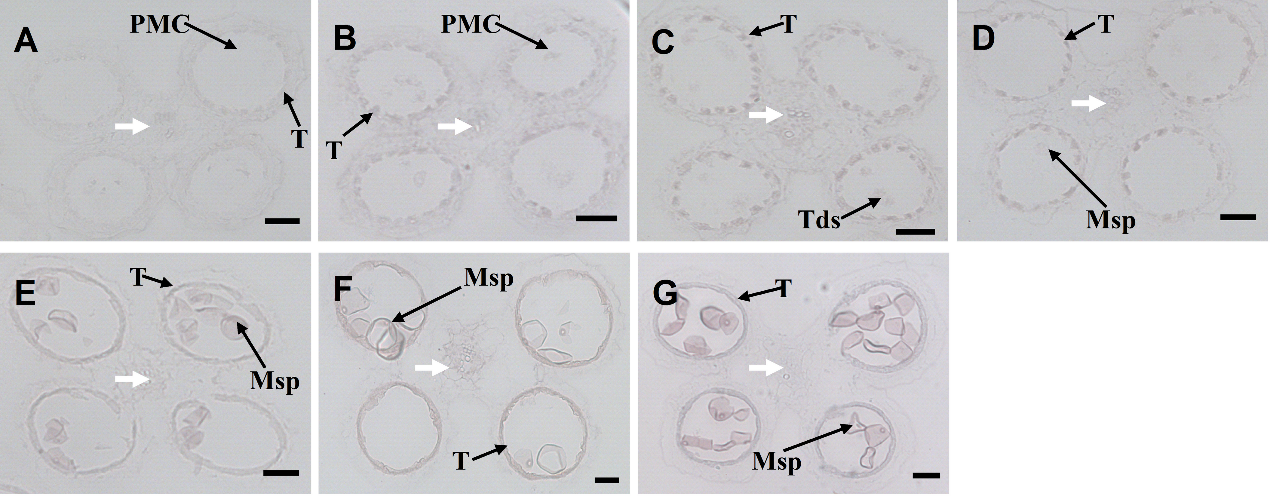
**

**Supplementary Fig. 5**  *In situ* RNA hybridization with an *OsABCG15* sense probe*.* (A–G) Stages 7-11, showing no visible signal in wild-type anthers with the sense probe. White arrows indicate the vascular connective tissue. PMC, pollen mother cell; Msp, microspore; T, tapetum; Tds, tetrads. Bars =25 μm

**Cover image legends**

**Cover image 1.** Wild-type stomatal apparatus of anther connective surface

**Cover image 2.** Mutant stomatal apparatus of anther connective surface

**Cover image 3**. Centrifugal growth of the wax in anther exo-surface

**Cover image 4.** Wild-type tetrads under scanning electron microscopy

**Cover image 5.** Tetrads under scanning electron microscopy
