# Supplementary material for: OsABCG15 encodes a membrane protein that plays an important role in anther cuticle and pollen exine formation in rice
Source: Plant Cell Rep. 2014 Aug 20;33(11):1881–99. doi: 10.1007/s00299-014-1666-8 (PMC4197380; doi:10.1007/s00299-014-1666-8)
Supplement: Supplementary file 7 — Supplementary material 7 (DOC 38 kb) [file 299_2014_1666_MOESM7_ESM.doc]

Supplementary Table 1 List of the primers used for mapping, RT-qPCR, RNA *in situ* analyses, and construction of the subcellular localization vector.

| **Primer name** | **Forward primer** | **Reverse primer** |
| --- | --- | --- |
| RM20356 | 5'TTACCAGGCTTCCTCTCTTGACC3' | 5'CCACGTCACCCAGAAACTAATCC3' |
| RM20361 | 5'CTTGAAATTTGTGCGGAGGTTGC3' | 5'GATGTCACCATCACGGAGAATTAGG3' |
| Chr6Ind(-30) | 5'GCTTCCATAACTACAAGGC3' | 5'ATCTCGTATAACAAACTCACAA3' |
| Chr6Ind(4) | 5'CGATATTACTACCAGGATTT3' | 5'ATTGCCAACCAACTAACT3' |
| Chr6STS20 | 5'AAAATGGTGGGTCATACGG3' | 5'GGGTCCTGGGTAGCGAAA3' |
| Chr6STS15 | 5'GCTCCGTGACCAAGTATGT3' | 5'GAGGAAGAAGAAGAGGGTGT3' |
| Chr6STS16 | 5'AGTATTTTGGAAGGACGGA3' | 5'CCCAGATATTATTTTAACCGA3' |
| Chr6STS13 | 5'CGATGAGATGGTTGGGAGC3' | 5'TGCGGGCAGGAGATTTGG3' |
| Chr6Ind(-7) | 5'TTACCAGGCTTCCTCTCTTGACC3' | 5'TTACCAGGCTTCCTCTCTTGACC3' |
| RM20366 | 5'CAGGTAAAGCGATGAGCAATTCG3' | 5'AAGGAGTTGGCAACAGCGAAGG3' |
| RM275 | 5'CCTCAACATCCTCACACACAAGC3' | 5'GCCAATCGGATGTGATTTATGC3' |
| RM5957 | 5'ACTGCTGCACTGCACAAGAC3' | 5'AGCTAGCTAGGCGTGAGCTG3' |
| RT-qPCRF/R | 5'GAGATCCTGGCACTGATG3' | 5'GTGTCACAAATCCAATCCTTCT3' |
| ActinF/R | 5'CTCTGTATGCCAGTGGTCGT3' | 5'CCGTTGTGGTGAATGAGTAAC3' |
| *In situ*  antisenseF/R | 5' GGAGATGATGGAGATCAGC 3' | 5'AGATTTAGGTGACACTATAGAATATCAG TGCCAGGATCTCAC3' |
| *In situ*  senseF/R | 5'AGTAATACGACTCACTATAGGGGGAGATGATGGAGATCAGC3' | 5' ATCAGTGCCAGGATCTCAC 3’ |
| GFP  fusionF/R | 5'CAGAGATCTATGATGGAGATCAGCA GCAATG3' | 5'ACCGGTACCCTACAAGGGCATGAGGCTGATCCGCTT3' |

Footnote: The boxed characters are SP6 and T7 promoter sequences in antisense and sense probe primers, respectively; the underlined characters are restriction sites. Abbreviations: Chr, chromosome; Ind, insert and delete.
