# Supplementary material for: OsABCG15 encodes a membrane protein that plays an important role in anther cuticle and pollen exine formation in rice
Source: Plant Cell Rep. 2014 Aug 20;33(11):1881–99. doi: 10.1007/s00299-014-1666-8 (PMC4197380; doi:10.1007/s00299-014-1666-8)
Supplement: Supplementary file 8 — Supplementary material 8 (DOC 41 kb) [file 299_2014_1666_MOESM8_ESM.doc]

Supplementary Table 2 The putative members of OsABCG15 family from other species

| **Accession** | **Species** | **Description** |
| --- | --- | --- |
| XP_003563648.1 | *Brachypodium distachyon* | ABC transporter G family member 26-like |
| XP_002438658.1 | *Sorghum bicolor* | hypothetical protein SORBIDRAFT_10g023750 |
| NP_001151511.1 | *Zea mays* | ABC transporter-like protein |
| XP_002438657.1 | *Sorghum bicolor* | hypothetical protein SORBIDRAFT_10g023740 |
| XP_002267511.2 | *Vitis vinifera* | ABC transporter G family member 26-like |
| XP_002884953.1 | *Arabidopsislyrata* | ABC transporter family protein |
| NP_187928.2 | *Arabidopsis thaliana* | ABC-2 type transporter family protein |
| XP_002522797.1 | *Ricinus communis* | ATP-binding cassette transporter, putative |
| XP_003552990.1 | *Glycine max* | ABC transporter G family member 26-like |
| XP_002300394.1 | *Populus trichocarpa* | white-brown-complex ABC transporter family |
| XP_003519640.1 | *Glycine max* | ABC transporter G family member 26-like |
| XP_003617868.1 | *Medicago truncatula* | ABC transporter G family member |
| XP_003544606.1 | *Glycine max* | ABC transporter G family member 26-like |
| XP_002992849.1 | *Selaginella moellendorffii* | hypothetical protein SELMODRAFT_136119 |
| XP_002969079.1 | *Selaginella moellendorffii* | ATP-binding cassette transporter |
| XP_001763976.1 | *Physcomitrella patens* | ABC transporter, subfamily G, PpABCG4 |
| XP_002322764.1 | *Populus trichocarpa* | white-brown-complex ABC transporter family |
| XP_003535833.1 | *Glycine max* | ABC transporter G family member 22-like |
| XP_003519092.1 | *Glycine max* | ABC transporter G family member 22-like |
| XP_002465834.1 | *Sorghum bicolor* | hypothetical protein SORBIDRAFT_01g046620 |
| XP_002263430.1 | *Vitis vinifera* | ABC transporter G family member 22-like |
| XP_003521505.1 | *Glycine max* | ABC transporter G family member 22-like |
| NP_001049014.1 | *Oryza sativa Japonica Group* | Os03g0157400 |
| XP_002524100.1 | *Ricinus communis* | ATP-binding cassette transporter, putative |
| XP_002873263.1 | *Arabidopsis lyrata* | ABC transporter family protein |
